# Functional Characterization of Four Olive Squalene Synthases with Respect to the Squalene Content of the Virgin Olive Oil

**DOI:** 10.1021/acs.jafc.3c05322

**Published:** 2023-10-10

**Authors:** M. Luisa Hernández, Cristina Muñoz-Ocaña, Pilar Posada, M. Dolores Sicardo, Dámaso Hornero-Méndez, Raquel B. Gómez-Coca, Angjelina Belaj, Wenceslao Moreda, José M. Martínez-Rivas

**Affiliations:** † Instituto de la Grasa (IG-CSIC), Campus Universitario Pablo de Olavide, Building 46, Ctra. Utrera Km.1, 41013 Sevilla, Spain; ‡ IFAPA Centro Alameda del Obispo, Avda. Menéndez Pidal s/n, 14080 Córdoba, Spain

**Keywords:** *Olea europaea*, olive fruit, core collection, SQS, gene expression, water stress

## Abstract

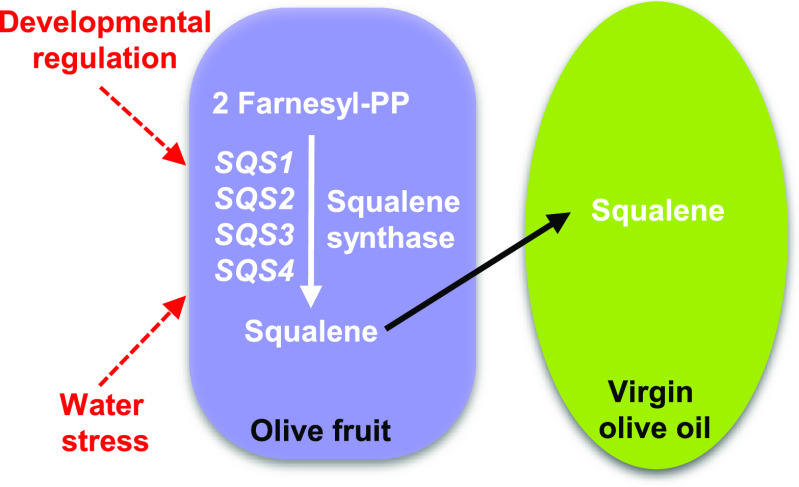

The release of new olive cultivars with an increased squalene content in their virgin olive oil is considered an important target in olive breeding programs. In this work, the variability of the squalene content in a core collection of 36 olive cultivars was first studied, revealing two olive cultivars, 'Dokkar' and 'Klon-14', with extremely low and high squalene contents in their oils, respectively. Next, four cDNA sequences encoding squalene synthases (SQS) were cloned from olive. Sequence analysis and functional expression in bacteria confirmed that they encode squalene synthases. Transcriptional analysis in distinct olive tissues and cultivars indicated that expression levels of these four *SQS* genes are spatially and temporally regulated in a cultivar-dependent manner and pointed to *OeSQS2* as the gene mainly involved in squalene biosynthesis in olive mesocarp and, therefore, in the olive oil. In addition, the biosynthesis of squalene appears to be transcriptionally regulated in water-stressed olive mesocarp.

## Introduction

Virgin olive oil (VOO) is obtained from olive fruit solely by physical procedures and with no preservatives or additives. World olive oil production and consumption have been rising considerably in the past few years not only because of its promising health benefits and remarkable nutritional properties^1^ but also due to its outstanding organoleptic characteristics. These properties are determined by the compounds present in the VOO. Specifically, VOO is constituted by triacylglycerols, which are the major components (≥98%), and a highly diverse mixture of chemical compounds called “minor compounds” (≤2%).^2^

Among the minor components present in the VOO, squalene is a polyunsaturated triterpenic hydrocarbon with six carbon double bonds. Due to its highly unsaturated chemical structure, the squalene molecule is sensitive to oxidation.^3^ In complex mixtures such as VOO, its stability is improved and, in turn, squalene was found to contribute to VOO stability under light exposure.^4^ However, the degradation of squalene in VOO after 6 months of storage in the dark has also been described.^5^ In addition, there is increasing evidence that squalene inhibits the thermal/oxidative degradation of frying oils.^6^

In recent years, special attention has been paid to squalene due to its potential health benefits of being included in the diet. The lower risk of cancer and cardiovascular diseases in the Mediterranean population has been related to the consumption of VOO, which contains significant amounts of squalene.^7^ In this regard, numerous studies have provided support for certain bioactivities of squalene such as antioxidant, anticancer, anti-inflammatory, and antiatherosclerosis, either *in vivo* or *in vitro*.^8^ Moreover, squalene is widely used in the pharmaceutical and cosmetic sector as a drug delivery agent, coadjuvant in vaccines, detoxifier, and skin protector.^9^

The main traditional source of squalene is shark liver oil, with a concentration reaching 40–86/100 g.^7^ However, the use of marine animal oil as a source of squalene has been limited by animal protection regulations and the presence of organic pollutants and heavy metals that cause cancer. For that reason, there is a general and increasing interest during the last decades in finding new sources of natural squalene, especially of plant origin.^7^ The highest content of squalene is observed in amaranth (6/100 g) and olive oils (0.5/100 g), although significant amounts are also present in other oils including wheat germ, rice bran, peanut, grape seed, and pumpkin seed.^3^ Of these plant species, squalene is recovered from the distillates of the olive oil industry.^10^

In plants, the biosynthesis of isoprenoids, which are the precursors of squalene, can be carried out via the mevalonate (MVA) pathway in the cytosol or through the 2C-methyl-d-erythritol-4-phosphate (MEP) pathway located in plastids.^11^ The MVA pathway generates only isopentenyl diphosphate (IPP), while the MEP pathway forms IPP and dimethylallyl diphosphate (DMAPP), with the isoprenoids exchange between cytosol and plastid being quite inefficient. Condensation of two molecules of IPP with DMAPP yielding farnesyl pyrophosphate (FPP) is catalyzed by FPP synthase (FPS). FPP is the substrate for the biosynthesis of phytosterols, ubiquinones, terpenoids, phytoalexins, and abscisic acid. Subsequently, squalene is synthesized by the condensation of two FPP molecules in a two-step reaction catalyzed by squalene synthase (SQS). In the first step, condensation of two molecules of FPP results in the formation of the intermediate presqualene diphosphate (PSPP), which is then reduced to squalene in the second step using NADPH as the substrate.^12^ Afterward, squalene can be converted to 2,3-oxidosqualene in a reaction catalyzed by squalene epoxidase (SQE), which is a key step in sterol biosynthesis.^13^ In **Arabidopsis thaliana**, two genes encoding SQS have been isolated and characterized, which show high sequence similarity.^14^ Both isoforms are located in the endoplasmic reticulum (ER), although only SQS1 displayed the expected enzymatic activity.^15^*SQS1* is expressed in all plant tissues, mainly in roots, whereas *SQS2* transcripts are especially abundant in leaves, cotyledons, and hypocotyls.

In addition to its role as a precursor of phytosterols and brassinosteroids, key molecules for plant growth and adaptation to biotic and abiotic stresses, squalene can regulate the biophysical properties, diffusion, and dynamic organization of cell membranes. Due to its nonpolar nature, squalene is localized on the hydrophobic center of the lipid bilayer, playing an important role in the electrochemical cell gradient.^3^

VOO consumption is the main source of squalene to cover its dietary needs.^7^ The squalene content in VOO ranges between 1.5 and 10.1 mg/g, and this amount is affected by genetic and agronomic factors including cultivar, fruit ripening, and agroclimatic conditions.^16^ Squalene does not undergo any relevant chemical or enzymatic transformation during the oil extraction process, being directly transferred from the olive fruit to the VOO. However, extraction technology and the refining process cause a considerable reduction in the squalene amount of olive oils.

Recently, the development of new olive cultivars producing VOO with improved nutritional quality, including the increase in the squalene content, has been considered a key goal in olive breeding programs.^17^ To achieve that, the identification of molecular markers associated with the high squalene content in VOO is needed. However, there is still little knowledge on the genetic control of its variability among olive cultivars, and molecular studies about squalene metabolism in olive fruit are lacking. In particular, olive *SQS* genes have not been cloned and characterized to date. Therefore, the objectives of our work were to analyze the squalene content in oils from the cultivars of a previously developed core collection (Core-36) with a wide genetic diversity^18^ to look for cultivars with high and low contents of squalene and the isolation and characterization of *SQS* genes in olive to determine the main candidates that could be responsible for the squalene content in the olive mesocarp and, therefore, in the olive oil. Specifically, functional identification and expression analysis during the development and ripening of olive fruit from different cultivars were performed not only to investigate their specific roles in squalene biosynthesis in distinct olive tissues but also to study the potential implication of *SQS* genes in response to water stress in the olive mesocarp.

## Materials and Methods

### Plant Materials

For oil analysis, olive (**Olea europaea** L.) fruit corresponding to a previously established core collection of 36 cultivars (Core-36)^18^ were harvested during the seasons 2011/2012 and 2012/2013. Olive trees from the Worldwide Olive Germplasm Bank of Córdoba (WOGBC) located at IFAPA (Alameda del Obispo, Córdoba, Spain) were cultivated by using standard culture practices. Since the WOGBC only possesses two trees for accession, the same sampling methodology used in previous studies performed with the Core-36^19^ was followed. In this sense, to obtain representative samples of the olive fruit from all parts of the olive trees, fruit were harvested by hand at the turning stage with a ripening index (RI) of 2.5 all around two trees per cultivar, mixed, and spliced into three different pools, which constitute three different biological samples.

For tissues and developmental studies, olive trees from the two main cultivars for oil production (cv. 'Picual' and 'Arbequina') were used. The trees were grown in the experimental orchard at Instituto de la Grasa (Seville, Spain), with drip irrigation and fertilization (irrigation with suitable fertilizers in the solution) from the time of full bloom to fruit maturation. Young drupes, developing seeds, and mesocarp tissue were harvested from at least three different olive trees at different weeks after flowering (WAF) corresponding to different developmental stages of the olive fruit: green (9, 16, and 19 WAF; RI 0); yellowish (23 WAF; RI 1); turning or veraison (28 and 31 WAF; RI 2 and 3, respectively); and mature or fully ripened (35 WAF; RI 4).

For mesocarp developmental studies of the selected cultivars with contrasting squalene content, olive trees (cv 'Dokkar' and 'Klon-14') from the WOGBC were used. Mesocarp tissue was harvested from two olive trees per each cultivar at different WAF corresponding to different developmental stages of the olive fruit: green (20 WAF; RI 0), yellowish (24 WAF; RI 1), turning or veraison (27 WAF; RI 2.5), and mature or fully ripen (31 WAF; RI 4).

To study the effect of different irrigation treatments, mesocarp samples at different WAF were harvested at the Sanabria orchard, a commercial super high-density olive (cv. 'Arbequina') orchard near Seville, as described by Hernández et al.^20^ Full irrigation (FI) and two regulated deficit irrigation (RDI) treatments (60RDI and 30RDI) were applied.

In all cases, olive tissues were frozen in liquid nitrogen immediately after harvest and stored at −80 °C.

### Olive Oil Extraction

Oil was extracted from olive fruit using a laboratory oil extraction plant (Abencor, Comercial Abengoa, SA. Seville, Spain), as described by Hernández et al.^19^

### Isolation of Olive Squalene Synthase Full-Length cDNA Clones and Sequence Analysis

Candidate olive *SQS* sequences were identified in the olive transcriptome^21^ and the wild olive (var. sylvestris) genome,^22^ using the tblastn algorithm as well as the amino acid sequences of Arabidopsis *SQS* genes. Based on these sequences, specific primers were designed for PCR amplification with ACCUZYME DNA polymerase (Bioline, Spain), which has proofreading activity. An aliquot of an olive Uni-ZAP XR cDNA library constructed with mRNA isolated from 13 WAF olive fruit of cultivar 'Picual' or cDNA obtained from leaves or mesocarp at the turning stage was used as a DNA template. One fragment of the expected size was generated in each reaction, subcloned into the vector pSpark I (Canvax, Spain), and sequenced in both directions by Sanger sequencing (GATC Biotech, Germany).

DNA sequence data were compiled and analyzed with the LASERGENE software package (DNAStar, Madison, WI, USA). The multiple sequence alignments of olive SQS deduced amino acid sequences were calculated by using the ClustalX program and displayed with GeneDoc. Phylogenetic tree analysis was performed using the neighbor-joining method implemented in the Phylip package using Kimura’s correction for multiple substitutions and a 1000 bootstrap data set. Treeview was used to display the tree. Subcellular localization was predicted by using three different programs: ProtComp (http://www.softberry.com), Wolf PSORT (http://wolfpsort.org/), and TargetP (http://www.cbs.dtu.dk/services/TargetP/), and the conserved domains of the deduced amino acid sequences of the *SQS* genes were detected using the Conserved Domain Database (CDD) search tool on the NCBI server (http://www.ncbi.nlm.nih.gov/structure/cdd/wrpsb.cgi). Hydropathy plots were generated by the method of Kyte and Doolittle (https://web.expasy.org/protscale/), and TMHMM analysis was also carried out (http://www.cbs.dtu.dk/services/TMHMM/).

### Total RNA Isolation and cDNA Synthesis

Total RNA isolation was performed as described by Hernández et al.^23^ using 1.5 g of the frozen olive tissue. RNA quality verification, removal of contaminating DNA, and cDNA synthesis were carried out according to Hernández et al.^24^

### Expression Analysis of Squalene Synthase Genes

Gene expression analysis was performed by quantitative real-time PCR (qRT-PCR) using a CFX connect real-time PCR system and iTaq Universal SYBR Green Supermix (BioRad, California, USA) as previously described.^25^ Primers for gene-specific amplification were designed using the Primer3 program (http://bioinfo.ut.ee/primer3/) and the Gene Runner program (Table S1). The housekeeping olive ubiquitin2 gene (*OeUBQ2*, AF429430) was used as an endogenous reference to normalize.^24^ For tissues and developmental studies of the different olive cultivars, the relative expression level of each gene was calculated using the 2^–ΔCt^ equation where ΔCt = (Ct_GOI_ – Ct_UBQ2_).^26,27^ This method has the advantage of making comparisons at the level of gene expression across developmental stages, cultivars, and genes. Regarding irrigation studies, the qRT-PCR data were calibrated relative to the corresponding gene expression level at 13 WAF from FI treatment as a calibrator. In these cases, the 2^–ΔΔCt^ method for relative quantification was followed.^26^ The data are presented as means ± SD of three biological replicates, each having two technical replicates per a 96-well plate.

### Functional Expression of Squalene Synthase Genes in **Escherichia coli**

Olive *SQS* coding sequences, except for the C-terminal retention signal to the ER in the case of *OepSQS1*, *OepSQS2*, and *OepSQS3* (28, 28, and 25 amino acids, respectively) or the N-terminal chloroplast signal peptide of *OepSQS4* (50 amino acids), were PCR-amplified using ACCUZYME DNA polymerase and specific primers with extended restriction enzyme sites for directional ligation (Table S2). The resulting PCR products were double-digested with the corresponding restriction enzymes and ligated under the control of the inducible T7*lac* promoter into bacterial expression vector pET45b(+) (Novagen, Germany). The cloning junctions were checked by sequencing before expression studies. The **E. coli** strain BL21 (DE3) (Novagen, Germany) was transformed with the resulting plasmids and selected on LB ampicillin plates. The LB medium containing ampicillin (6 mL) was inoculated with a single colony and grown at 37 °C until the *A*_600_ was 0.3–0.5. Then, 100 mL of the LB ampicillin medium was inoculated with the whole preculture and grown at 37 °C. When the culture reached an *A*_600_ of 0.6, 100 mM isopropyl-d-1-thiogalactopyranoside (IPTG) was added to induce gene expression, and the culture was further incubated at 22 °C for 20 h. Bacterial cells were harvested by centrifugation at 2,500*g* for 10 min at 4 °C, washed with 50 mM phosphate buffer pH 7.5, frozen in liquid nitrogen, and kept at −80 °C.

To obtain the crude extract, the frozen cell pellet was thawed and resuspended in 5 mL of extraction buffer [50 mM phosphate buffer pH 7.5, 1 mM dithiothreitol (DTT), and 1 mM phenylmethylsulfonyl fluoride] and disrupted by sonication. The lysate was centrifuged at 13,000*g* for 15 min at 4 °C, and the supernatant was used as a crude extract. The protein content was determined using the BioRad Bradford protein reagent dye with bovine serum albumin as a standard.

### *In Vitro* Assay of SQS Activity

The SQS activity was assayed as described by Ye et al.^28^ with modifications. An aliquot of the crude extract corresponding to 25 μg of protein was incubated in a total volume of 700 μL with 50 mM phosphate buffer pH 7.5, 2.5 mM NADPH, 10 mM DTT, 5 mM MgCl_2_, and 25 μM farnesyl pyrophosphate triammonium salt (FPP). After 2 h at 35 °C, the reaction mixture was extracted three times with 400 μL of hexane. Squalane (10 μg) was used as the internal standard. Samples were evaporated under a N_2_ stream and resuspended in 50 μL of hexane for squalene determination by GC.

### Squalene Determination

For squalene analysis in olive oil, the procedure described by Lanzón et al.^29^ was followed with modifications. The olive oil sample (40 mg) together with 20 μL of 10 mg/mL squalane as the internal standard (response coefficient equaled 1) was dissolved in 1 mL of hexane with vortexing and saponified at room temperature for 10 min with 200 μL of 2N KOH–MeOH. Two phases appear, and after washing the upper part with 3 × 400 μL of ethanol:water 1:1 (v/v) solution, 1 μL of the upper phase was used for GC.

For squalene analysis in olive tissues, the procedure described by Fernández-Cuesta et al.^30^ was followed with modifications. The lyophilized tissue (200 mg of mesocarp, seed, or leaf) together with 20 μL of 10 mg/mL squalane as the internal standard and 2 mL of 2% KOH-EtOH was heated at 80 °C for 15 min for alkaline hydrolysis. The unsaponifiable fraction was extracted by vortexing with 1 mL of hexane and 1.5 mL of distilled water. The upper hexane layer was transferred to a new tube and concentrated under a N_2_ stream before GC analysis.

The GC analysis was performed with an Agilent 7890A gas chromatograph fitted with an HP-5 column (30 m length; 0.32 mm internal diameter; 0.25 μm film thickness; Agilent, Santa Clara, CA, USA) and a flame ionization detector. Hydrogen was used as a carrier gas at a linear rate of 4.96 mL/min and a split ratio of 1:20. The injector and detector temperatures were 300 and 325 °C, respectively, and the oven temperature was 250 °C for 3 min and subsequently was raised to 290 °C at a 4 °C/min rate.

Squalene identification from the *in vitro* SQS activity assay was performed by GC-mass spectrometry (GC-MS) on a GC Finnigan Trace-GC 2000 coupled to a Polaris-Q ion trap mass spectrometer (ThermoFinnigan, Austin, TX, USA), which operates in full scan mode. Chromatographic analysis was carried out using a DB-5MS capillary column (30 m length; 0.25 mm internal diameter; 0.25 μm film thickness) (Agilent). Helium was used as a carrier gas at a constant flow rate of 1.5 mL/min and a split ratio of 1:20. The column temperature was maintained at 250 °C for 3 min and then elevated to 270 °C at 4 °C/min for 25 min. The injector temperature was 300 °C, and the ion trap heating was 200 °C.

## Results and Discussion

### Variability of the Squalene Content in an Olive Cultivar Core Collection (Core-36)

**Figure 1:**
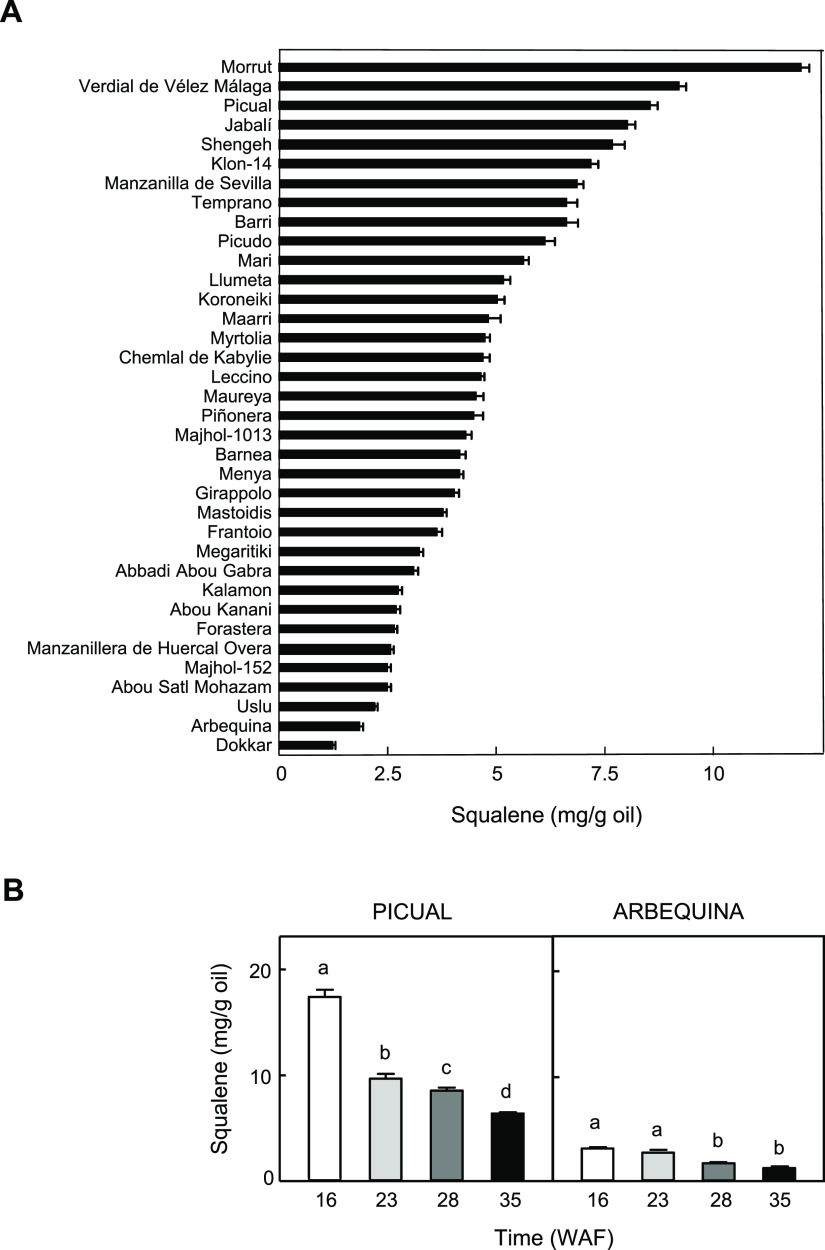
Squalene content in the oils of cultivars from the Core-36 extracted from olive fruit harvested at the turning stage with a ripening index of 2.5 (A) or from 'Picual' and 'Arbequina' cultivars extracted from olive fruit at different developmental stages: green (16 WAF), yellowish (24 WAF), turning (28 WAF), and mature (31 WAF) (B). Oil extraction and squalene analysis were performed as described in the Materials and Methods section. Data are presented as means ± SD of three biological replicates. Different letters denote significant differences (*P* < 0.05) for 'Picual' or 'Arbequina' cultivar by one-way ANOVA followed by Tukey’s post-test for multiple comparisons.

The squalene content of a Core-36,^18^ which holds most of the genetic diversity found at the WOGBC, was first analyzed to identify olive cultivars with contrasting squalene contents. The mean percentage of squalene was 4.52 mg/g oil, and the variability intervals ranged from 1.27 to 11.83 mg/g oil, with the cultivars 'Dokkar' and 'Morrut' being the ones with the most contrasting squalene content (Figure 1A). These values show a high level of variability and are similar to those reported for another set of 28 cultivars from the same germplasm collection,^16^ confirming a high level of variability for the squalene content among cultivars and representing new opportunities for breeding. In Italian and Tunisian cultivars, the amounts of squalene varied from 3.5 to 5.8 and from 1.4 to 5.4 mg/g oil, respectively.^5,31^ Interestingly, the variability ranges described in the present study were slightly lower than those observed for the squalene content in the segregating progeny of the cross between 'Picual' and 'Arbequina' cultivars, which ranged from 2.5 to 8.5 mg/g oil.^17^ A high level of variability of the Core-36 has also been previously described for the fatty acid profile.^19^ Our results also support the evidence that cultivar differences in the squalene content of olive oils are mainly due to the genetic component as previously reported,^16^ since all the olive cultivars were grown in the same orchard and growing conditions, olive fruits were harvested at the same ripening stage, and identical oil extraction conditions were used. Therefore, these data further confirm that the genotype is the main source of variability for the squalene content in olive oil.

In addition, a continuous decrease in the squalene content was detected in olive oils extracted from 'Picual' and 'Arbequina' fruit during development and ripening (Figure 1B). A similar pattern has been reported for Tunisian cultivars,^31^ although the kinetics of this process seems to be cultivar-dependent.

### cDNA Cloning and Sequence Analysis of Four Olive Squalene Synthase Genes

**Figure 2:**
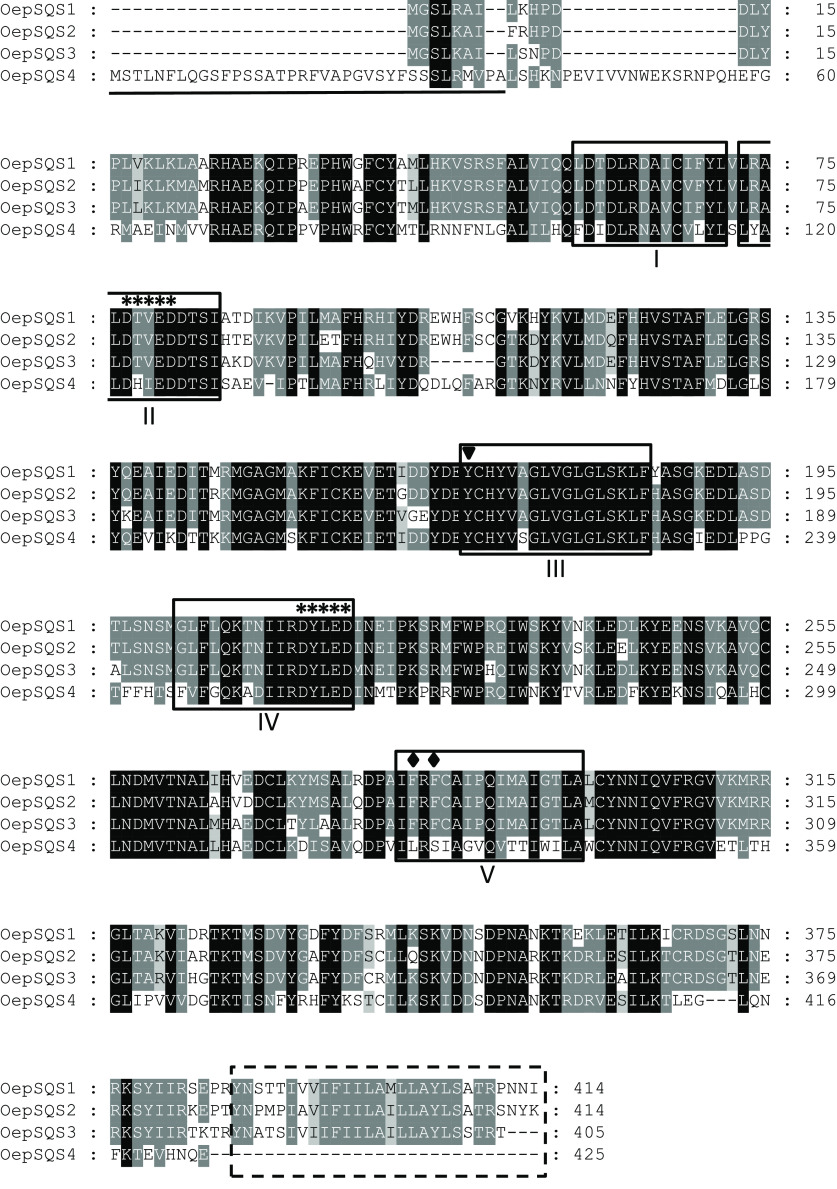
Comparison of the deduced amino acid sequences of olive *SQS* genes. The sequences were aligned using the ClustalX program and displayed with GeneDoc. Identical and similar residues are shown on a background of black and gray, respectively. The putative chloroplast transit peptide is indicated by a solid line. Conserved domains characteristic of SQS genes (I–V) are boxed with a solid line. The two Asp-rich motifs (DXXXD), which are involved in the binding of FPP, are labeled with asterisks. The conserved Tyr and two Phe residues are denoted by an inverted triangle and two rhombi, respectively. The hydrophobic C-terminal region is framed with a dashed line. The cDNA sequences corresponding to *OepSQS1,OepSQS2*, *OepSQS3*, and *OepSQS4* have been deposited in the GenBank/EMBL/DDBJ database with accession numbers OQ676921, OQ676922, OQ676923, and OQ676924, respectively.

Four sequences were identified from the olive transcriptome^21^ and the wild olive (var. sylvestris) genome,^22^ which displayed an elevated degree of similarity to the Arabidopsis *SQS1* gene.^14^ Based on these sequences, specific primer pairs were designed and utilized for PCR amplification, along with an aliquot of an olive fruit (13 WAF) cDNA library (cv. 'Picual') or cDNA obtained from leaves or mesocarp at the turning stage. The four full-length cDNA clones were isolated and named *OepSQS1, OepSQS2, OepSQS3*, and *OepSQS4*, with sizes of 1555, 1454, 1562, and 1264 bp, respectively. They exhibited ORFs encoding predicted proteins of 414, 414, 405, and 425 amino acid residues, which correspond to calculated molecular masses of 47.7, 47.6, 46.2, and 48.9 kDa, respectively, and p*I* values of 8.0 for OepSQS1, 7.6 for OepSQS2, 8.0 for OepSQS3, and 6.9 for OepSQS4. Alignment of the four olive SQS deduced amino acid sequences (Figure 2) showed that OepSQS1 displayed 86 and 85% identity with respect to OepSQS2 and OepSQS3, respectively, whereas these last two shared 84% identity. In contrast, OepSQS4 shares low identity with the rest of the sequences, displaying 47, 48, and 46% identity with OepSQS1, OepSQS2, and OepSQS3, respectively.

NCBI database search for conserved domains revealed that the four proteins belong to the isoprenoid biosynthesis class 1 superfamily. Pfam analysis showed the presence of a squalene/phytoene synthase domain (Pfam: 00494) between amino acids 44-315, 44-315, 44-309, and 92-341 for OepSQS1, OepSQS2, OepSQS3, and OepSQS4, respectively.

Five highly conserved domains were identified in the alignment of the SQS deduced amino acid sequences (Figure 2). Domains III, IV, and V showed highly conserved sequences to those from other plant SQS, whereas domains I and II were less conserved.^32^ These highly conserved domains are considered important for catalytic/functional activity. Domain I is the chemical binding site or substrate binding pocket. Domain II and IV contain two Asp-rich motifs (DXXXD) shown in Figure 2, which are considered to coordinate and facilitate the binding of FPP by binding Mg^2+^ ions.^33^ Domain III is the active site, and the first Tyr residue in this region (Figure 2) is essential for the first step of catalysis because mutations in this amino acid result in a complete loss of SQS activity.^34^ Finally, domain V is the NADPH binding region (Figure 2) and contains two conserved Phe.^35^ Accordingly, domains I, II, III, and IV are involved in the first half reaction, the condensation of two molecules of FPP to PSPP, while domain V is required for the conversion of PSPP into squalene, being essential for the second half reaction.^12^

Transmembrane protein topology analyses using hidden Markov model (TMHMM) software revealed that OepSQS4 does not contain any transmembrane domain (TMD), while olive SQS1, SQS2, and SQS3 possess two TMD composed of 22 amino acid residues each, both in the C-terminal region of the protein (Figure S1). This topology strongly indicates that olive SQS1, SQS2, and SQS3 are membrane-bound proteins and is in agreement with the topology reported for plant SQS proteins, where one or two TMD have been described with the same C-terminal location.^35^ The second TMD is highly hydrophobic (Figure S2) with low sequence similarity (Figure 2) and is presumably responsible for the anchor of the SQS protein to the ER membrane.^36^ In line with this notion, Busquets et al.^15^ reported the target of a GFP version of AtSQS1 to the ER membrane in onion epidermal cells and demonstrated that this targeting depends solely on the presence of the second TMD.

Analysis of the deduced olive SQS protein sequences with subcellular localization prediction software such as ProtComp or WoLF PSORT suggests that OepSQS1, OepSQS2, and OepSQS3 could be located in the ER, while OepSQS4 could be localized in the chloroplast. In addition, an N-terminal transit peptide with the characteristic features of chloroplast targeting peptides was detected by TargetP software in the OepSQS4 sequence (Figure 2), with a predicted cleavage site after Glu at residue 50. Experimental localization for plant SQS protein not only has been mainly reported in the ER^15,37−39^ but also has been located in the cytoplasm,^39,40^ nucleus,^39^ and plasma membrane.^35^

An unrooted phylogenetic tree based on deduced amino acid sequences of known and characterized plant SQS (Figure S3) was generated to investigate the phylogenetic relationship of olive SQS. In agreement with previous findings,^41^ phylogenetic analysis showed that SQS sequences consisted of several distinct branch clusters that accompanied species divergence, which could be grouped into four lineages: Pteridophytes, Gymnosperms, Monocotyledons, and Eudicotyledons. The relationship displayed in the phylogenetic tree thus corresponded to their taxonomic classifications. Olive SQS sequences were positioned very close between them, in a clade together with other SQS from dicot plants. Interestingly, OepSQS4 was placed at a far evolutionary distance in relation to the other plant SQS.

### Functional Expression of Olive Squalene Synthase Genes in **E. coli**

**Figure 3:**
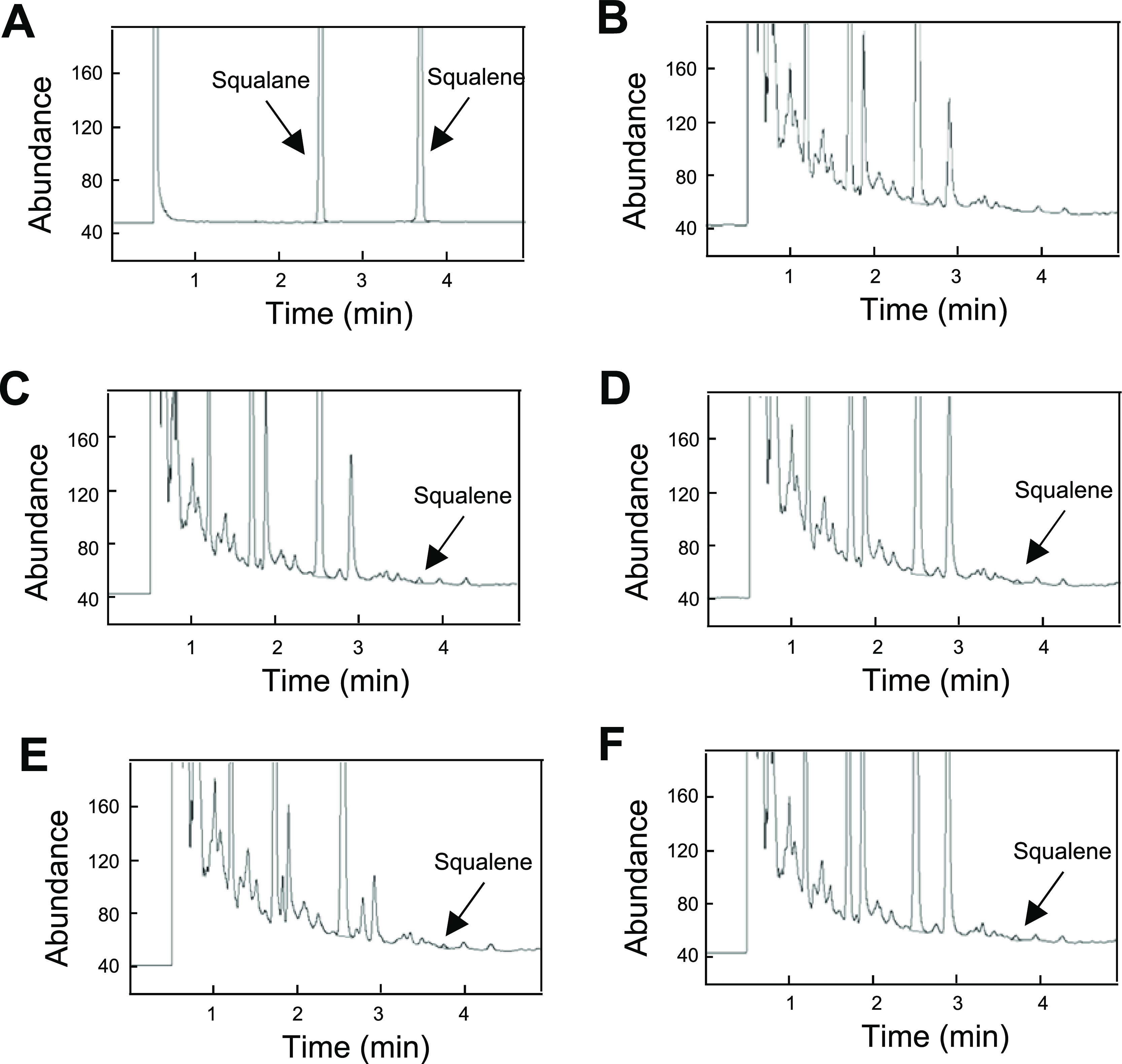
GC analysis of authentic squalane (internal standard) and squalene (external standard) (A) and from the reaction products of the SQS activity assay containing protein extracts from **E. coli** culture without IPTG (B) or after the induction of the expression of OepSQS1 (C), OepSQS2 (D), OepSQS3 (E), and OepSQS4 (F). The reaction products were extracted from the *in vitro* reaction mixture and analyzed by GC as described in the Materials and Methods section.

To confirm the functional identity of the four olive *SQS* genes, the corresponding coding regions, excluding the sequences of the C-terminal ER retention signal for OepSQS1, OepSQS2, and OepSQS3 or the N-terminal chloroplast signal peptide in the case of OepSQS4 (Figure 2), were placed under the control of an IPTG-inducible promoter of an *E. coli* expression vector. It was reported that the removal of the C-terminal hydrophobic region could enhance the soluble expression of recombinant SQS.^41^ Bacterial cells containing the four olive *SQS* overexpression constructs grown at 22 °C for 20 h expressed the corresponding proteins. To verify that these four polypeptides were products of the *SQS* gene, the resultant bacterial crude extracts were used to perform the SQS *in vitro* activity assay. The reaction product analysis carried out by GC showed the presence of a new peak with a retention time identical to that of squalene, which was absent in the control activity assay without IPTG induction (Figure 3). GC-MS analysis demonstrated that the novel peak corresponds to squalene (Figure S4). Therefore, the four olive *SQS* genes have been functionally identified since they code for the enzyme that catalyzed the synthesis of squalene from two molecules of FPP in the presence of NADPH and Mg^2+^.

### Tissue Specificity of Olive Squalene Synthase Genes

**Figure 4:**
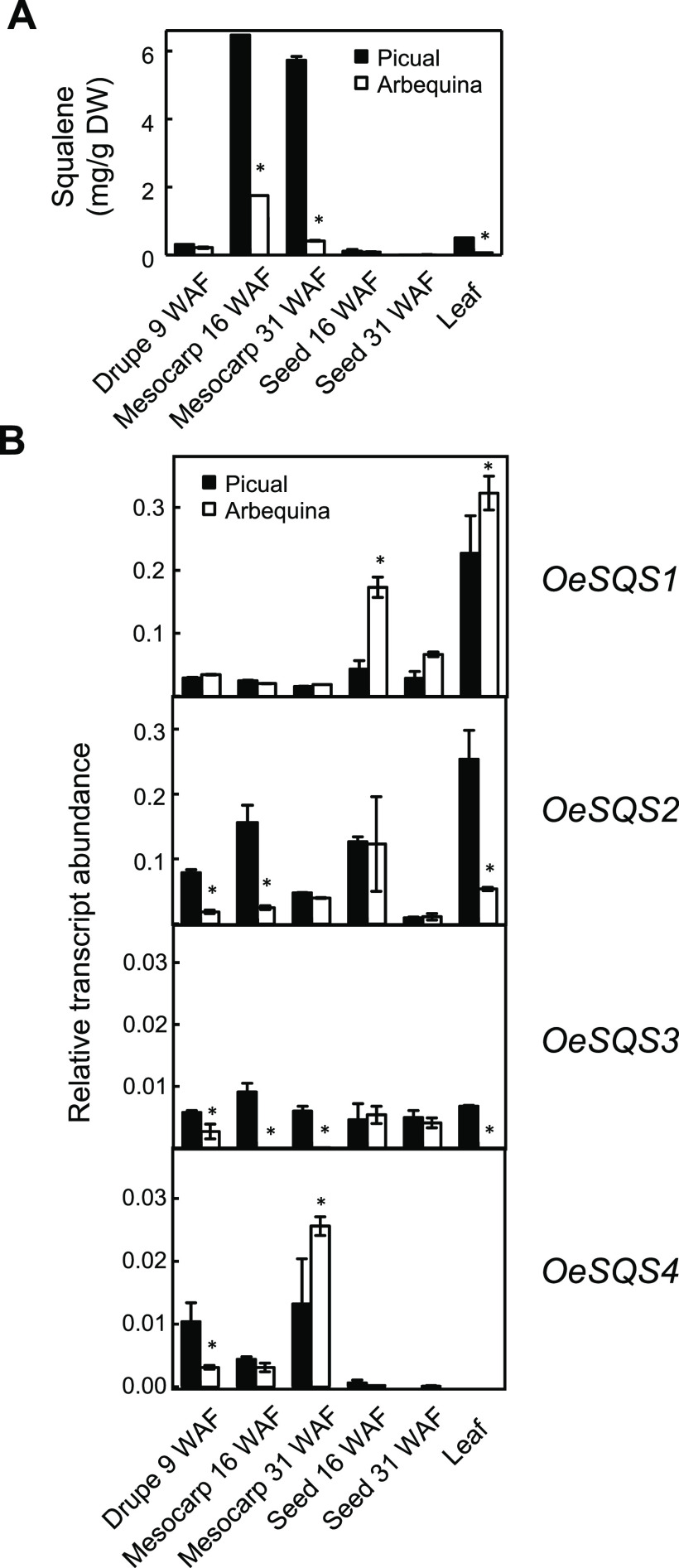
Squalene content (A) and relative transcript abundance of olive *SQS* genes (B) in different tissues of 'Picual' and 'Arbequina' cultivars. The amount of squalene was quantified by GC, and the relative transcript abundance was determined by qRT-PCR in the indicated tissues as described under the Materials and Methods section. Data are presented as means ± SD of three biological replicates. Asterisk indicates significantly different (*P* < 0.05) to 'Picual' by two-way analysis of variance (ANOVA) with a Bonferroni post-test in 'Arbequina' tissues.

To investigate the physiological function of the four olive *SQS* genes, we analyzed the squalene content and the *SQS* transcript levels in distinct olive organs and tissues from the two main cultivars for oil production: 'Picual' and 'Arbequina' (Figure 4). Very low levels of squalene were found in young drupes, developing seeds, or leaves (Figure 4A). In contrast, the mesocarp showed the highest amount of squalene compared with the rest of the tissues, with their contents being higher in 'Picual' than in 'Arbequina' cultivar (Figure 4A).

As shown in Figure 4B, *OeSQS1* and *OeSQS2* exhibited considerably higher expression levels than did *OeSQS3* and *OeSQS4* in all analyzed tissues. In particular, *OeSQS1* showed the highest transcript levels in 'Arbequina' young seeds and leaves from both cultivars, whereas *OeSQS2* was highly expressed in 'Picual' and 'Arbequina' young seeds as well as in leaves, young drupes, and mesocarp from 'Picual' cultivar. The high expression levels of olive *SQS1* and *SQS2* detected in leaves could be related to the accumulation of phytosterol and triterpenes in this tissue since no correlation between *SQS* transcript levels and squalene content was found in olive leaves (Figure 4). Concerning *OeSQS3,* low expression levels were found in all studied tissues, except in the case of 'Arbequina' mesocarp and leaves, where no transcript was detected. Finally, *OeSQS4* transcripts were observed in young drupes and mesocarp, being almost undetectable in seeds and leaves. All these data indicate a spatial regulation of *SQS* genes in olive, considering that they were differentially expressed in all studied organs and tissues.

The *SQS* tissue expression pattern varies greatly and depends on the species. *SQS* high transcript levels have been detected in young tissues like the leaves of **Betula platyphylla**,^40^ in organs containing dividing cells such as the roots of leguminous plants as in the case of **Medicago sativa**,^35^ and in organs rich in phytosterols and/or triterpenoids like in the roots of medicinal plants including ginseng.^42^

### Developmental Expression of Squalene Synthase Genes in the Olive Fruit in Relation to the Squalene Content of the Virgin Olive Oil

**Figure 5:**
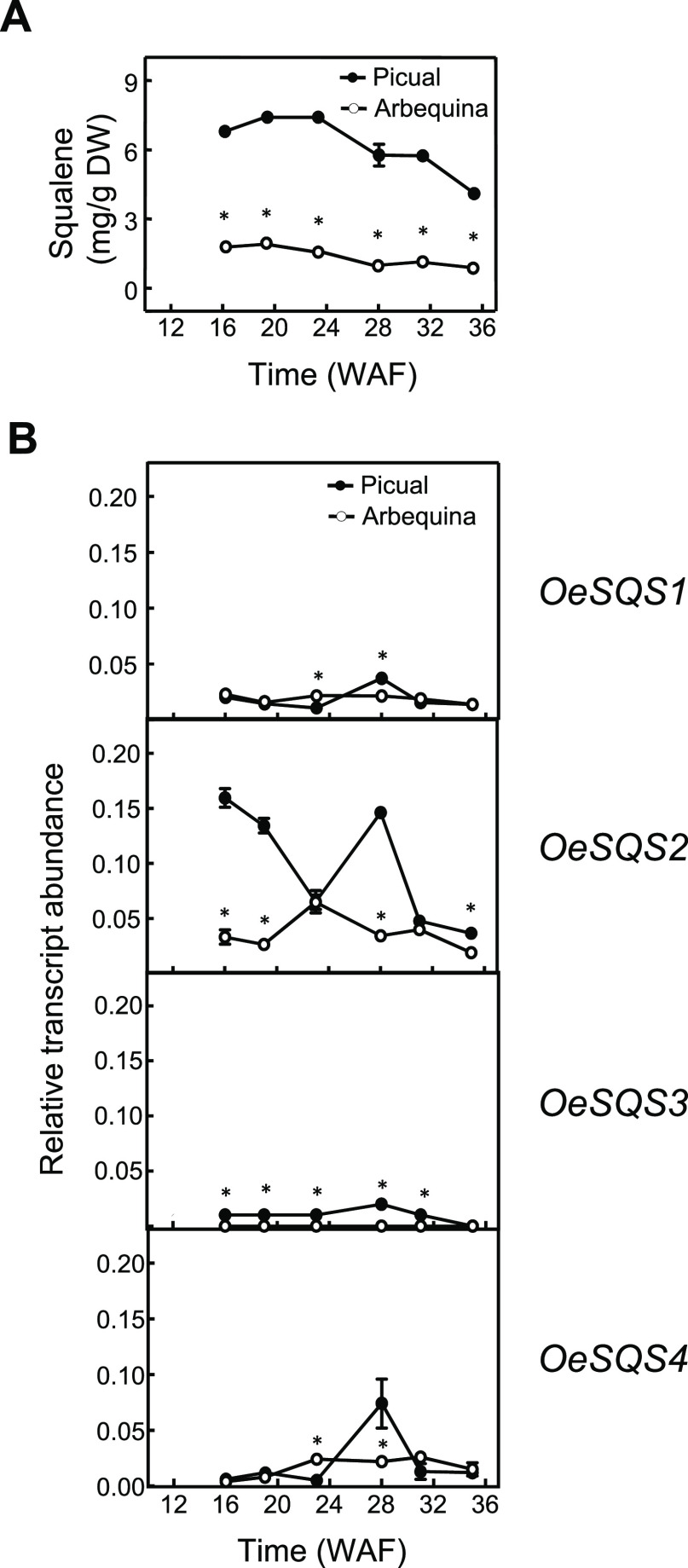
Squalene content (A) and relative transcript abundance of olive *SQS* genes (B) in the mesocarp tissue of 'Picual' and 'Arbequina' cultivars during the olive fruit development and ripening. At the indicated times, the amount of squalene was quantified by GC and the relative transcript abundance was determined by qRT-PC as described under the Materials and Methods section. Data are presented as means ± SD of three biological replicates. Asterisk indicates significantly different (*P* < 0.05) to 'Picual' by two-way analysis of variance (ANOVA) with a Bonferroni post-test in 'Arbequina'.

The squalene content of the 'Picual' and 'Arbequina' seeds (Figure S5A) was very much lower than that of the mesocarp (Figure 5A). In concordance with the data obtained using olive oils (Figure 1B), the amount of squalene was higher in the mesocarp from 'Picual' compared to 'Arbequina' (Figure 5A). This was expected since the contribution of the mesocarp tissue to the final composition of the olive oil is much higher than that of the seed.^43^

A progressive reduction in the squalene content was also observed in the seed and mesocarp tissues of both cultivars during fruit development and ripening (Figure S5A and Figure 5A). Similar results were reported from other cultivars such as 'Chemlali' and 'Oueslati'.^44^ This reduction of squalene in olive fruit can be attributed not only to its conversion to other compounds such as sterols and triterpenes but also to its antioxidant role in oxidative reactions that started in the matured olive fruit.^31^

Interestingly, the diminution detected in the tissues of 'Picual' and 'Arbequina' cultivars was not as strong as that observed in the corresponding olive oils (Figure 1B). This fact can be explained because the concentration of squalene in the oils depends not only on the decreased squalene content in olive fruit but also on the dilution effect due to the increased oil accumulation in the fruit.^30^

Next, the expression levels of olive *SQS* genes in the olive fruit, from which the VOO is obtained, were studied in more detail to identify which one is mainly responsible for the squalene content in the VOO. Specifically, seeds and mesocarp tissues during the development and ripening of olive fruit from the cultivars 'Picual' and 'Arbequina' were analyzed.

Regarding developing seeds (Figure S5B), *OeSQS4* transcript levels were almost undetectable in both cultivars, while *OeSQS1*, *OeSQS2*, and *OeSQS3* genes showed higher expression levels. In 'Picual', these three *SQS* genes showed a peak at 23 WAF, whereas in the case of 'Arbequina', a decrease in *OeSQS1* and *OeSQS2* transcripts was detected at early stages of development as well as a constant and very low level in the case of *OeSQS3* during the whole developmental and ripening period.

Expression of *SQS* genes in seeds has also been reported from other plants such as tea, ginseng, and soybean.^38,42,45^ In the case of amaranth seeds, which accumulate great amounts of squalene, low levels of *SQS* transcripts were observed at the initial stages of development, increasing rapidly at the mid-late developmental stage before decreasing at the late stages.^46^

A similar investigation was performed in olive mesocarp (Figure 5B). The expression analysis of *SQS* genes in the mesocarp of 'Picual' and 'Arbequina' cultivars showed that the highest transcript levels were observed for the *OeSQS2* gene, and these levels were higher in 'Picual' mesocarp than those in 'Arbequina'. Furthermore, the expression pattern of the *OeSQS2* gene during mesocarp development and ripening was different in both cultivars. In 'Picual', *OeSQS2* showed high transcript levels at the beginning of fruit development with a reduction, followed by a peak at 28 WAF (turning stage), and another decrease at the end of the ripening period. In the case of 'Arbequina', a more continuous expression level was observed throughout the developmental and ripening period. Regarding olive *SQS1*, *SQS3*, and *SQS4* genes, all exhibited low and steady transcript levels in both cultivars, apart from *OeSQS4* in 'Picual' at 28 WAF where a peak is detected. All of these data point out that in olive fruit, the expression of *SQS* genes seems to be temporally regulated during the developmental and ripening period. In the case of other fruits, a diminution of *SQS* transcript levels has been described during the development and ripening of persimmon fruit.^47^

In olive, the higher expression level detected for *OeSQS2* in 'Picual' mesocarp is in line with the higher squalene content observed for this cultivar compared to 'Arbequina'. This result, together with the fact that this gene showed the highest transcript level of olive *SQS* genes in the mesocarp tissue, indicates that *OeSQS2* seems to be the gene that mainly contributes to the biosynthesis of squalene in the olive mesocarp.

To confirm this hypothesis, our study was expanded to other olive cultivars characterized by a low ('Dokkar') and high ('Klon-14') squalene content (Figure 1A) using mesocarp tissues corresponding to four different representative stages of fruit development and ripening (Figure S6). As observed for 'Picual' and 'Arbequina', olive *SQS1*, *SQS3*, and *SQS4* genes in 'Dokkar' and 'Klon-14' cultivars showed very low transcript levels compared to *OeSQS2* (Figure S6B), confirming that this gene is the main responsible for the squalene biosynthesis in the olive mesocarp. However, a clear correlation between the squalene content (Figure S6A) and the *OeSQS2* transcript levels during the development and ripening of 'Dokkar' and 'Klon-14' fruit was not found. These data suggest that not only SQS but other enzymes, such as squalene epoxidase that converts squalene in 2,3-oxidosqualene prior to sterol biosynthesis,^13^ could be involved in determining the squalene content in olive mesocarp and, therefore, in the olive oil.

### Effect of Regulated Deficit Irrigation on Squalene Synthase Gene Expression in the Olive Fruit Mesocarp

The effect of different water regimes on the squalene content of olive oil has been previously studied with dissimilar results depending on the cultivar. An increased squalene content was reported for olive oils obtained in rain-fed conditions in the case of the 'Chétoui' cultivar.^48^ On the contrary, the squalene content was reduced in oils from 'Barnea' and 'Souri' olive trees receiving the lowest irrigation rates.^49^ In the present study, the squalene content in the mesocarp tissue was lower in 'Picual' and 'Arbequina' fruit cultivated in rain-fed conditions, especially in the case of 'Picual' (Figure S7).

**Figure 6:**
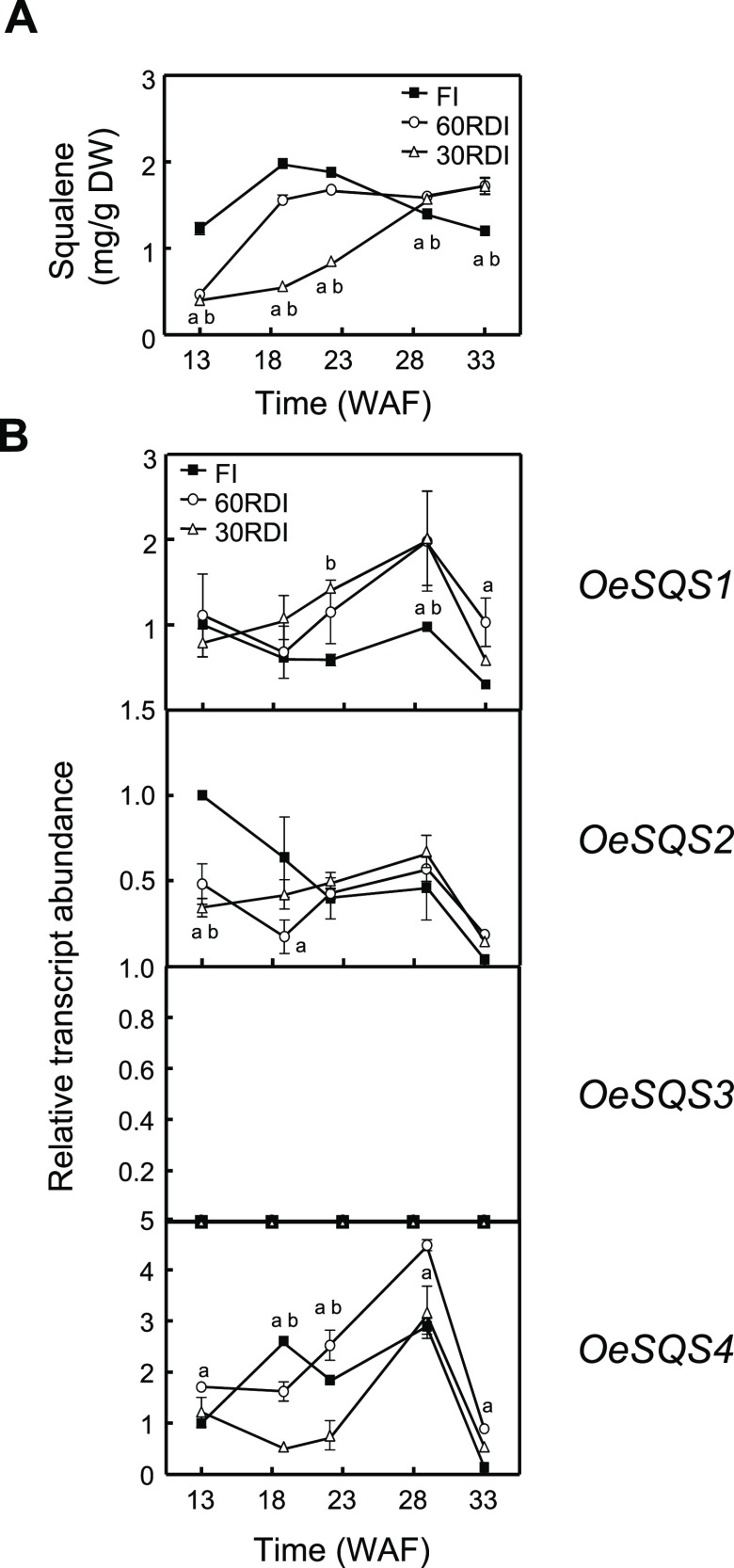
Effect of regulated deficit irrigation treatments on the squalene content (A) and the relative transcript abundance of olive *SQS* genes (B) in the mesocarp tissue from cultivar 'Arbequina' during olive fruit development and ripening. At the indicated times, the amount of squalene was quantified by GC and the relative transcript abundance was determined by qRT-PC as described under the Materials and Methods section. Data are presented as means ± SD of three biological replicates. Superscripts a and b indicate significantly different (*P* < 0.05) in 60RDI and 30RDI, respectively, to FI by two-way analysis of variance (ANOVA) with a Bonferroni post-test.

In earlier studies by our group,^20^ the effect of three distinct RDI treatments on the oil accumulation, fatty acid profile, and fatty acid desaturase gene expression levels in 'Arbequina' fruit mesocarp was investigated. However, data related to the effect of water stress on the squalene content and expression levels of olive genes involved in squalene biosynthesis have not yet been reported. In this study, a reduction in the squalene content of the mesocarp at green (13–19 WAF) and yellowish (22 WAF) stages of fruit development was observed in 'Arbequina' mesocarp from olives subjected to 30RDI and 60RDI treatments, which produced substantial levels of water stress, in comparison to FI treatment (Figure 6A). In agreement with these results, *OeSQS2* lower expression levels were detected at the green stages in fruit under deficit irrigation (Figure 6B), further confirming the main role of this gene in the biosynthesis of squalene in the mesocarp of the olive fruit. In contrast, *OeSQS1* showed a continuous upregulation of transcript levels in water-stressed 'Arbequina' mesocarp, which correlates with the sustained increase in the squalene content observed in the fruit under the same treatments during development and ripening. On the other hand, *OeSQS3* and *OeSQS4* genes exhibited very low and constant expression levels or changes in those, which do not correlate well with the amount of squalene in the corresponding water-stressed fruit, respectively.

Therefore, the biosynthesis of squalene appears to be transcriptionally regulated in water-stressed olive mesocarp, with *OeSQS1* and *OeSQS2* genes increasing and decreasing their expression levels at the end and beginning of fruit development, respectively. As a result, the amount of squalene decreased in 'Arbequina' mesocarp under deficit irrigation at the green stage because of *OeSQS2* down-regulation and then recovered due to an increase in *OeSQS1* transcripts to reach a squalene content similar to that observed in full irrigated mesocarp during the ripening period.

A down-regulation of *SQS* gene expression levels has been reported in soybean plants under water-deficit conditions,^45^ which is in agreement with the negative effects on drought adaptation reported in rice for SQS when constitutive silencing of rice SQS using an RNAi approach resulted in improved water-deficit tolerance.^50^ On the contrary, an increase in *SQS* transcript levels has been described in other plants under drought stress, such as apple tree leaves.^37^ In water stress conditions, it has been suggested that *SQS* expression could be induced to potentially change membrane composition since phytosterol and triterpene contents impact membrane fluidity and stability depending on osmotic fluctuation.^37^

In conclusion, the squalene content exhibits a high degree of variability in the olive oils from the Core-36 olive cultivar collection. In addition, the isolation and characterization of four olive *SQS* genes have been performed. Sequence analysis of these genes (*OepSQS1*, *OepSQS2*, *OepSQS3*, and *OepSQS4*) shows that they code for SQS enzymes. The identity of the *SQS* genes was confirmed by the functional expression in bacteria. Gene expression analysis reveals spatial and temporal regulation of olive *SQS* transcript levels in olive fruit in the course of development and ripening and points out that olive *SQS* gene expression is cultivar-dependent. In addition, transcriptional data from 'Picual' and 'Arbequina', together with those obtained from two cultivars with a highly contrasted squalene content such as 'Dokkar' (low) and 'Klon-14' (high), suggest that *OeSQS2* seems to be the gene that mainly contributes to the biosynthesis of squalene in the olive mesocarp and, therefore, in the olive oil. The expression of *OeSQS1* and *OeSQS2* genes in 'Arbequina' olive fruit is regulated by water status, preserving the squalene content at the ripening stage independently of the water regime. This study represents substantial progress in the knowledge of the regulation of squalene biosynthesis in olive fruit. In addition, this information will allow the generation of molecular markers for the marker-assisted selection of new olive cultivars with an increased squalene content in the VOO.

## Supporting Information Available

Predicted TMD for olive SQS sequences (Figure S1), hydropathy plot of the olive SQS predicted amino sequences (Figure S2), phylogenetic tree analysis of plant squalene synthases (Figure S3), mass spectrum of the peak for authentic squalene and squalene synthesized by olive SQS (Figure S4), squalene content and relative transcript abundance of olive *SQS* genes in the seed tissue of 'Picual' and 'Arbequina' cultivars (Figure S5), squalene content and relative transcript abundance of olive *SQS* genes in the mesocarp tissue of 'Dokkar' and 'Klon-14' cultivars (Figure S6), effect of water regime on the squalene content of the mesocarp tissue (Figure S7), sequences of the primer pairs used for gene expression analysis by qRT-PCR (Table S1), sequences of the primer pairs used for amplification of olive *SQS* coding sequences for their functional expression in *E. coli* (Table S2), and accession numbers of the different SQS included in the phylogenetic tree analysis (Table S3) (PDF)

## References

[ref1] Escrich E.; Ramírez-Tortosa M. C.; Sánchez-Rovira P.; Colomer R.; Solanas M.; Gaforio J. J. Olive oil in cancer prevention and progression. Nutr. Rev. 2006, 64, 40–52. 10.1301/nr.2006.oct.S40-S52.

[ref2] Aparicio R.; Harwood J. L. (2013). Handbook of olive oil: analysis and properties; 2nd ed.; Springer: New York, USA, 2013.

[ref3] Lozano-Grande M. A.; Gorinstein S.; Espitia-Rangel E.; Dávila-Ortiz G.; Martínez-Ayala A. L. Plant sources, extraction methods, and uses of squalene. Int. J. Agron. 2018, 1829160 10.1155/2018/1829160.

[ref4] Psomiadou E.; Tsimidou M. Stability of virgin olive oil. 2. Photo-oxidation studies. J. Agric. Food Chem. 2002, 50, 722–727. 10.1021/jf010847u. 11829635

[ref5] Manzi P.; Panfili G.; Esti M.; Pizzoferrato L. Natural antioxidants in the unsaponifiable fraction of virgin olive oils from different cultivars. J. Sci. Food Agric. 1998, 77, 115–120. 10.1002/(SICI)1097-0010(199805)77:1<115::AID-JSFA13>3.0.CO;2-N.

[ref6] Chiou A.; Kalogeropoulos N.; Salta F. N.; Efstathiou P.; Andrikopoulos N. K. Pan-frying of French fries in three different edible oils enriched with olive leaf extract: Oxidative stability and fate of microconstituents. LWT--Food Sci. Technol. 2009, 42, 1090–1097. 10.1016/j.lwt.2009.01.004.

[ref7] Naziri E.; Mantzouridou F.; Tsimidou M. Z. Squalene resources and uses point to the potential of biotechnology. Lipid Technol. 2011, 23, 270–273. 10.1002/lite.201100157.

[ref8] Micera M.; Botto A.; Geddo F.; Antoniotti S.; Bertea C. M.; Levi R.; Gallo M. P.; Querio G. Squalene: more than a step toward sterols. Antioxidants 2020, 9, 688 10.3390/antiox9080688. 32748847 PMC7464659

[ref9] Kim S.-K.; Karadeniz F. Biological importance and applications of squalene and squalane. Adv. Food Nutr. Res. 2012, 65, 223–233. 10.1016/B978-0-12-416003-3.00014-7. 22361190

[ref10] Bondioli P.; Mariani C.; Lanzani A.; Dedeli E.; Muller A. Squalene recovery from olive oil deodorizer distillates. J. Am. Oil Chem. Soc. 1993, 70, 763–766. 10.1007/BF02542597.

[ref11] Lichtenthaler H. K.; Rohmer M.; Schwender J. Two independent biochemical pathways for isopentenyl diphosphate and isoprenoid biosynthesis in higher plants. Physiol. Plant. 1997, 101, 643–652. 10.1111/j.1399-3054.1997.tb01049.x.

[ref12] Pandit J.; Danley D. E.; Schulte G. K.; Mazzalupo S.; Pauly T. A.; Hayward C. M.; Hamanaka E. S.; Thompson J. F.; Harwood H. J. Crystal structure of human squalene synthase. A key enzyme in cholesterol biosynthesis. J. Biol. Chem. 2000, 275, 30610–30617. 10.1074/jbc.M004132200. 10896663

[ref13] Spanova M.; Daum G. Squalene - biochemistry, molecular biology, process biotechnology, and applications. Eur. J. Lipid Sci. Technol. 2011, 113, 1299–1320. 10.1002/ejlt.201100203.

[ref14] Kribii R.; Arró M.; del Arco A.; González V.; Balcells L.; Delourme D.; Boronat A. Cloning and characterization of the *Arabidopsis thaliana SQS1* gene encoding squalene synthase. Involvement of the C-terminal region of the enzyme in the channeling of squalene through the sterol pathway. Eur. J. Biochem. 1997, 249, 61–69. 10.1111/j.1432-1033.1997.00061.x. 9363754

[ref15] Busquets A.; Keim V.; Closa M.; del Arco A.; Boronat A.; Arró M.; Ferrer A. *Arabidopsis thaliana* contains a single gene encoding squalene synthase. Plant Mol. Biol. 2008, 67, 25–36. 10.1007/s11103-008-9299-3. 18236008

[ref16] Beltrán G.; Bucheli M. E.; Aguilera M. P.; Belaj A.; Jiménez A. Squalene in virgin olive oil: screening of variability in olive cultivars. Eur. J. Lipid Sci. Techn. 2016, 118, 1250–1253. 10.1002/ejlt.201500295.

[ref17] Velasco L.; Fernandez-Cuesta A.; de la Rosa R.; Ruiz-Mendez M. V.; Leon L. Selection for some olive oil quality components through the analysis of fruit flesh. J. Am. Oil Chem. Soc. 2014, 91, 1731–1736. 10.1007/s11746-014-2523-1.

[ref18] Belaj A.; Dominguez-Garcia M. C.; Atienza S. G.; Martin-Urdiroz N.; de la Rosa R.; Satovic Z.; Martin A.; Kilian A.; Trujillo I.; Valpuesta V.; del Rio C. Developing a core collection of olive (*Olea europaea* L.) based on molecular markers (DArTs, SSRs, SNPs) and agronomic traits. Tree Genet. Genomes 2012, 8, 365–378. 10.1007/s11295-011-0447-6.

[ref19] Hernández M. L.; Sicardo M. D.; Belaj A.; Martínez-Rivas J. M. The oleic/linoleic acid ratio in olive (*Olea europaea* L.) fruit mesocarp is mainly controlled by *OeFAD2–2* and *OeFAD2–5* genes together with the different specificity of extraplastidial acyltransferase enzymes. Front. Plant Sci. 2021, 12, 653997 10.3389/fpls.2021.653997. 33763103 PMC7982730

[ref20] Hernández M. L.; Velázquez-Palmero D.; Sicardo M. D.; Fernández J. E.; Diaz-Espejo A.; Martínez-Rivas J. M. Effect of a regulated deficit irrigation strategy in a hedgerow “Arbequina” olive orchard on the mesocarp fatty acid composition and desaturase gene expression with respect to olive oil quality. Agric. Water Manag. 2018, 204, 100–106. 10.1016/j.agwat.2018.04.002.

[ref21] Muñoz-Mérida A.; González-Plata J. J.; Cañada A.; Blanco A. M.; García-López M. C.; Rodríguez J. M.; et al. *De novo* assembly and functional annotation of the olive (*Olea europaea*) transcriptome. DNA Res. 2013, 20, 93–108. 10.1093/dnares/dss036. 23297299 PMC3576661

[ref22] Unver T.; Wu Z.; Sterck L.; Turktas M.; Lohaus R.; Li Z.; Van de Peer Y.; et al. Genome of wild olive and the evolution of oil biosynthesis. Proc. Natl. Acad. Sci. U. S. A. 2017, 114, 9413–9422. 10.1073/pnas.1708621114. PMC5676908 29078332

[ref23] Hernández M. L.; Mancha M.; Martínez-Rivas J. M. Molecular cloning and characterization of genes encoding two microsomal oleate desaturases (*FAD2*) from olive. Phytochemistry 2005, 66, 1417–1426. 10.1016/j.phytochem.2005.04.004. 15896817

[ref24] Hernández M. L.; Padilla M. N.; Mancha M.; Martínez-Rivas J. M. Expression analysis identifies *FAD2–2* as the olive oleate desaturase gene mainly responsible for the linoleic acid content in virgin olive oil. J. Agric. Food Chem. 2009, 57, 6199–6206. 10.1021/jf900678z. 19601663

[ref25] Hernández M. L.; Sicardo M. D.; Alfonso M.; Martínez-Rivas J. M. Transcriptional regulation of stearoyl-acyl carrier protein desaturase genes in response to abiotic stresses leads to changes in the unsaturated fatty acids composition of olive mesocarp. Front. Plant Sci. 2019, 10, 251 10.3389/fpls.2019.00251. 30891055 PMC6411816

[ref26] Livak K. J.; Schmittgen T. D. Analysis of relative gene expression data using real-time quantitative PCR and the 2^–ΔΔCt^ method. Methods 2001, 25, 402–408. 10.1006/meth.2001.1262. 11846609

[ref27] Pfaffl M. W. Quantification strategies in real-time PCR. In A-Z. of Quantitative PCR; Bustin S. A., Ed.; International University Line: La Jolla, USA, 2004; pp 87–112.

[ref28] Ye Y.; Wang R.; Jin L.; Shen J.; Li X.; Yang T.; Chen Y. Molecular cloning and differential expression analysis of a squalene synthase gene from *Dioscorea zingiberensis*, an important pharmaceutical plant. Mol. Biol. Rep. 2014, 41, 6097–6104. 10.1007/s11033-014-3487-9. 24996285

[ref29] Lanzón A.; Guinda A.; Albi T.; de la Osa C. A rapid method for squalene determination in vegetable oils. Grasas Aceites 1995, 46, 276–278. 10.3989/gya.1995.v46.i4-5.937.

[ref30] Fernandez-Cuesta A.; Leon L.; Velasco L.; de la Rosa R. Changes in squalene and sterols associated with olive maturation. Food Res. Int. 2013, 54, 1885–1889. 10.1016/j.foodres.2013.07.049.

[ref31] Laroussi-Mezghani S.; Le Dréau Y.; Molinet J.; Hammami M.; Grati-Kamoun N.; Artaud J. Biodiversity of Tunisian virgin olive oils: varietal origin classification according to their minor compounds. Eur. Food Res. Technol. 2016, 242, 1087–1099. 10.1007/s00217-015-2613-9.

[ref32] Vishwakarma R. K.; Patel K.; Sonawane P.; Kumari U.; Singh S.; Ruby; Abbassi S.; Agrawal D. C.; Tsay H.-S.; Khan B. M. Squalene synthase gene from medicinal herb *Bacopa monniera*: molecular characterization, differential expression, comparative modeling, and docking studies. Plant Mol. Biol. Rep. 2015, 33, 1675–1685. 10.1007/s11105-015-0864-z.

[ref33] Huang Z.; Jiang K.; Pi Y.; Hou R.; Liao Z.; Cao Y.; Han X.; Wang Q.; Sun X.; Tang K. Molecular cloning and characterization of the yew gene encoding squalene synthase from *Taxus cuspidata*. J. Biochem. Mol. Biol. 2007, 40, 625–635. 10.5483/BMBRep.2007.40.5.625. 17927893

[ref34] Gu P.; Ishii Y.; Spencer T. A.; Shechter I. Function–structure studies and identification of three enzyme domains involved in the catalytic activity in rat hepatic squalene synthase. J. Biol. Chem. 1998, 273, 12515–12525. 10.1074/jbc.273.20.12515. 9575210

[ref35] Kang J.; Zhang Q.; Jiang X.; Zhang T.; Long R.; Yang Q.; Wang Z. Molecular cloning and functional identification of a squalene synthase encoding gene from alfalfa (*Medicago sativa* L.). Int. J. Mol. Sci. 2019, 20, 4499 10.3390/ijms20184499. 31514406 PMC6770234

[ref36] Robinson G. W.; Tsay Y. H.; Kienzle B. K.; Smith-Monroy C. A.; Bishop R. W. Conservation between human and fungal squalene synthetases: similarities in structure, function, and regulation. Mol. Cell. Biol. 1993, 13, 2706–2717. 10.1128/mcb.13.5.2706-2717.1993. 8474436 PMC359645

[ref37] Navarro Gallón S. M.; Elejalde-Palmett C.; Daudu D.; Liesecke F.; Jullien F.; Papon N.; et al. Virus-induced gene silencing of the two squalene synthase isoforms of apple tree (*Malus* x *domestica* L.) negatively impacts phytosterol biosynthesis, plastid pigmentation and leaf growth. Planta 2017, 246, 45–60. 10.1007/s00425-017-2681-0. 28349256

[ref38] Fu J. Y.; Liu G. H.; Yang M.; Wang X. C.; Chen X. L.; Chen F.; Yang Y. J. Isolation and functional analysis of squalene synthase gene in tea plant *Camellia sinensis*. Plant Physiol. Biochem. 2019, 142, 53–58. 10.1016/j.plaphy.2019.06.030. 31272035

[ref39] Wu J.; Xu R.; Lu J.; Liu W.; Yu H.; Liu M.; Li J.; Yin M.; Peng H.; Zha L. Molecular cloning and functional characterization of two squalene synthase genes in *Atractylodes lancea*. Planta 2022, 255, 8 10.1007/s00425-021-03797-9. 34845523

[ref40] Zhang M.; Wang S.; Yin J.; Li C.; Zhan Y.; Xiao J.; Liang T.; Li X. Molecular cloning and promoter analysis of squalene synthase and squalene epoxidase genes from *Betula platyphylla*. Protoplasma 2016, 253, 1347–1363. 10.1007/s00709-015-0893-3. 26464187

[ref41] Zha L. P.; Liu S.; Su P.; Yuan Y.; Huang L. Q. Cloning, prokaryotic expression and functional analysis of squalene synthase (SQS) in *Magnolia officinalis*. Protein Expression Purif. 2016, 120, 28–34. 10.1016/j.pep.2015.12.008. 26696600

[ref42] Lee M. H.; Jeong J. H.; Seo J. W.; Shin C. G.; Kim Y. S.; In J. G.; Choi Y. E. Enhanced triterpene and phytosterol biosynthesis in *Panax ginseng* overexpressing squalene synthase gene. Plant Cell Physiol. 2004, 45, 976–984. 10.1093/pcp/pch126. 15356323

[ref43] Hernández M. L.; Sicardo M. D.; Martínez-Rivas J. M. Differential contribution of endoplasmic reticulum and chloroplast ω-3 fatty acid desaturase genes to the linolenic acid content of olive (*Olea europaea*) fruit. Plant Cell Physiol. 2016, 57, 138–151. 10.1093/pcp/pcv159. 26514651

[ref44] Ben Mansour A.; Flamini G.; Ben Selma Z.; Le Dreau Y.; Artaud J.; Abdelhedi R.; Bouaziz M. Olive oil quality is strongly affected by cultivar, maturity index and fruit part: Chemometrical analysis of volatiles, fatty acids, squalene and quality parameters from whole fruit, pulp and seed oils of two Tunisian olive cultivars. Eur. J. Lipid Sci. Technol. 2015, 117, 976–987. 10.1002/ejlt.201400159.

[ref45] Nguyen H. T. M.; Neelakadan A. K.; Quach T. N.; Valliyodan B.; Kuma R.; Zhang Z.; Nguyen H. T. Molecular characterization of *Glycine max* squalene synthase genes in seed phytosterol biosynthesis. Plant Physiol. Biochem. 2013, 73, 23–32. 10.1016/j.plaphy.2013.07.018. 24036394

[ref46] Park Y.-J.; Nemoto K.; Minami M.; Matsushima K. Molecular cloning, expression and characterization of a squalene synthase gene from grain amaranth (*Amaranthus cruentus* L.). Jpn. Agric. Res. Q. 2016, 50, 307–317. 10.6090/jarq.50.307.

[ref47] Zhou C.; Zhao D.; Sheng Y.; Liang G.; Tao J. Molecular cloning and expression of squalene synthase and 2,3-oxidosqualene cyclase genes in persimmon (*Diospyros kaki* L.) fruits. Mol. Biol. Rep. 2012, 39, 1125–1132. 10.1007/s11033-011-0841-z. 21573791

[ref48] Baccouri O.; Guerfel M.; Baccouri B.; Cerretani L.; Bendini A.; Lercker G.; Zarrouk M.; Ben Miled D. D. Chemical composition and oxidative stability of Tunisian monovarietal virgin olive oils with regard to fruit ripening. Food Chem. 2008, 109, 743–754. 10.1016/j.foodchem.2008.01.034. 26049987

[ref49] Ben-Gal A.; Dag A.; Basheer L.; Yermiyahu U.; Zipori I.; Kerem Z. The influence of bearing cycles on olive oil quality response to irrigation. J. Agric. Food Chem. 2011, 59, 11667–11675. 10.1021/jf202324x. 21950468

[ref50] Manavalan L. P.; Chen X.; Clarke J.; Salmeron J.; Nguyen H. T. RNAi-mediated disruption of squalene synthase improves drought tolerance and yield in rice. J. Exp. Bot. 2012, 63, 163–175. 10.1093/jxb/err258. 21926092 PMC3245457

