# Supplementary material for: Functional Characterization of Four Olive Squalene Synthases with Respect to the Squalene Content of the Virgin Olive Oil
Source: J Agric Food Chem. 2023 Oct 10;71(42):15701–12. doi: 10.1021/acs.jafc.3c05322 (PMC10723762; doi:10.1021/acs.jafc.3c05322)
Supplement: Supplementary file 1 — jf3c05322_si_001.pdf [file jf3c05322_si_001.pdf]

# Functional Characterization of Four Olive Squalene Synthases with Respect to the Squalene Content of the Virgin Olive Oil

M. Luisa Hernández[1a], Cristina Muñoz-Ocaña[1], Pilar Posada[1], M. Dolores Sicardo[1], Dámaso Hornero-Méndez[1], Raquel B. Gómez-Coca[1], Angjelina Belaj[2], Wenceslao Moreda[1], and José M. Martínez-Rivas[1,*]

[1]Instituto de la Grasa (IG-CSIC), Campus Universitario Pablo de Olavide, Building 46, Ctra. Utrera Km.1, 41013 Sevilla, Spain.

[2]IFAPA Centro Alameda del Obispo, Avda. Menéndez Pidal s/n 14080, Córdoba, Spain

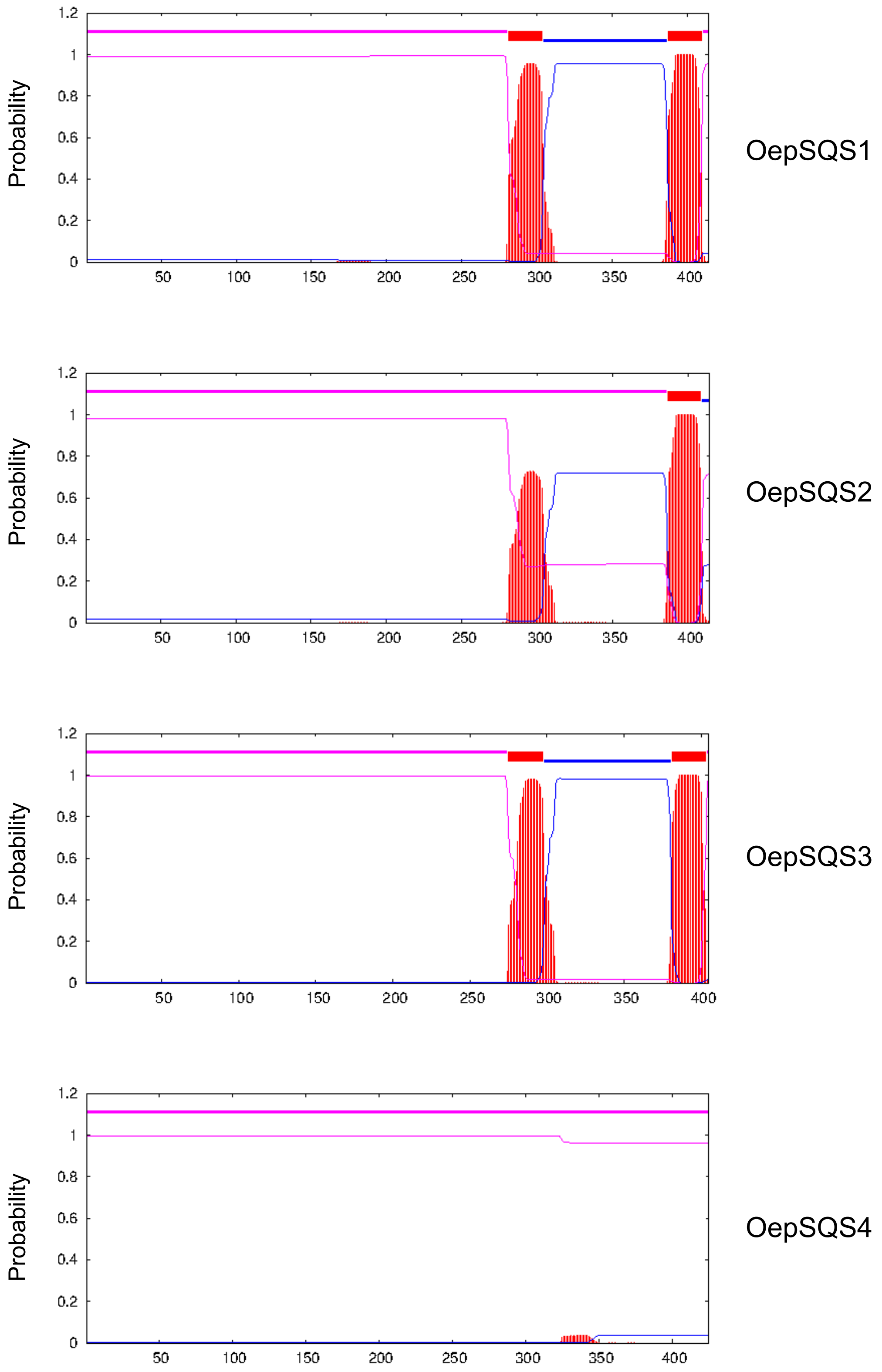


**Figure S1.** Predicted transmembrane domains for olive SQS sequences. Predicted transmembrane helix (shown in red) were identified by TMHMM analysis for OepSQS1 and OepSQS2, OepSQS3, and OepSQS4. Regions of the olive SQS sequences predicted to be located inside or outside the membrane are shown in blue and pink, respectively.

OepSQS1 OepSQS2

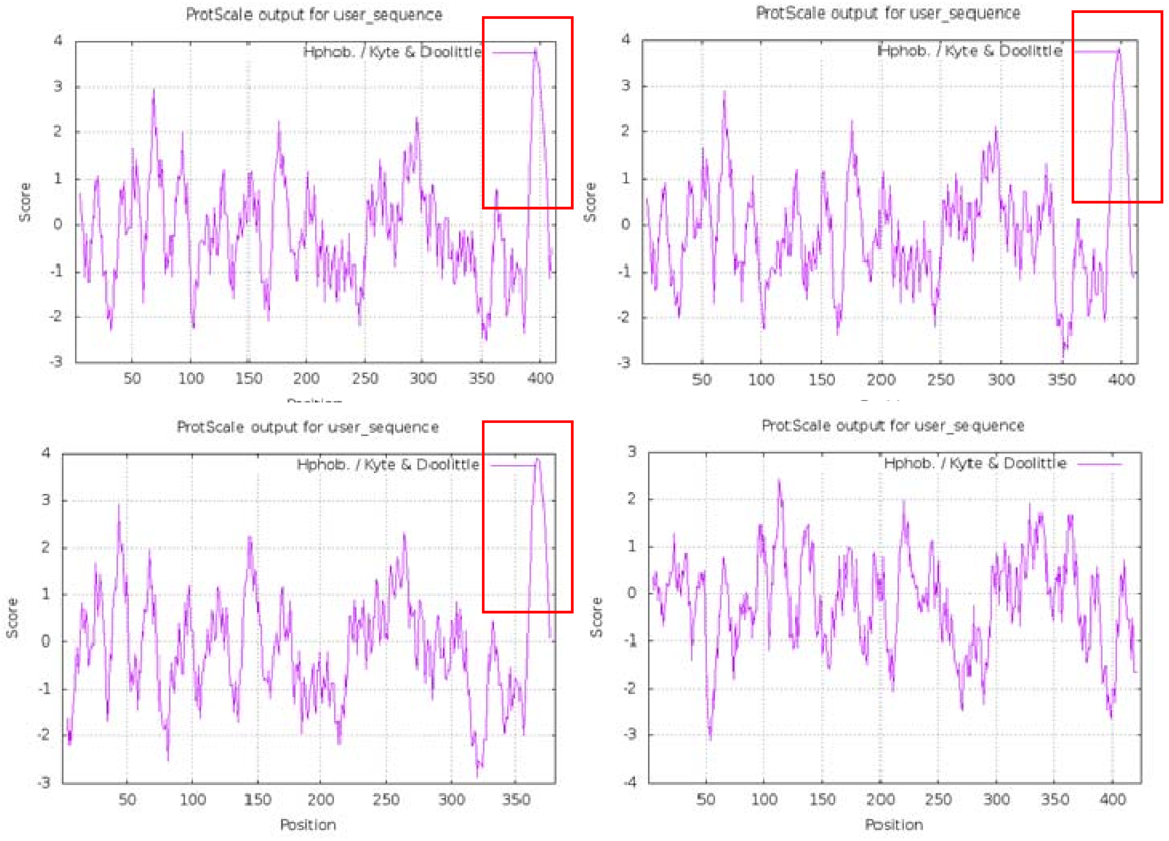


OepSQS3 OepSQS4

**Figure S2.** Hydropathy plot of the olive SQS predicted amino sequences generated by the method of Kyte and Doolittle. C-terminal hydrophobic regions are indicated in red squares.

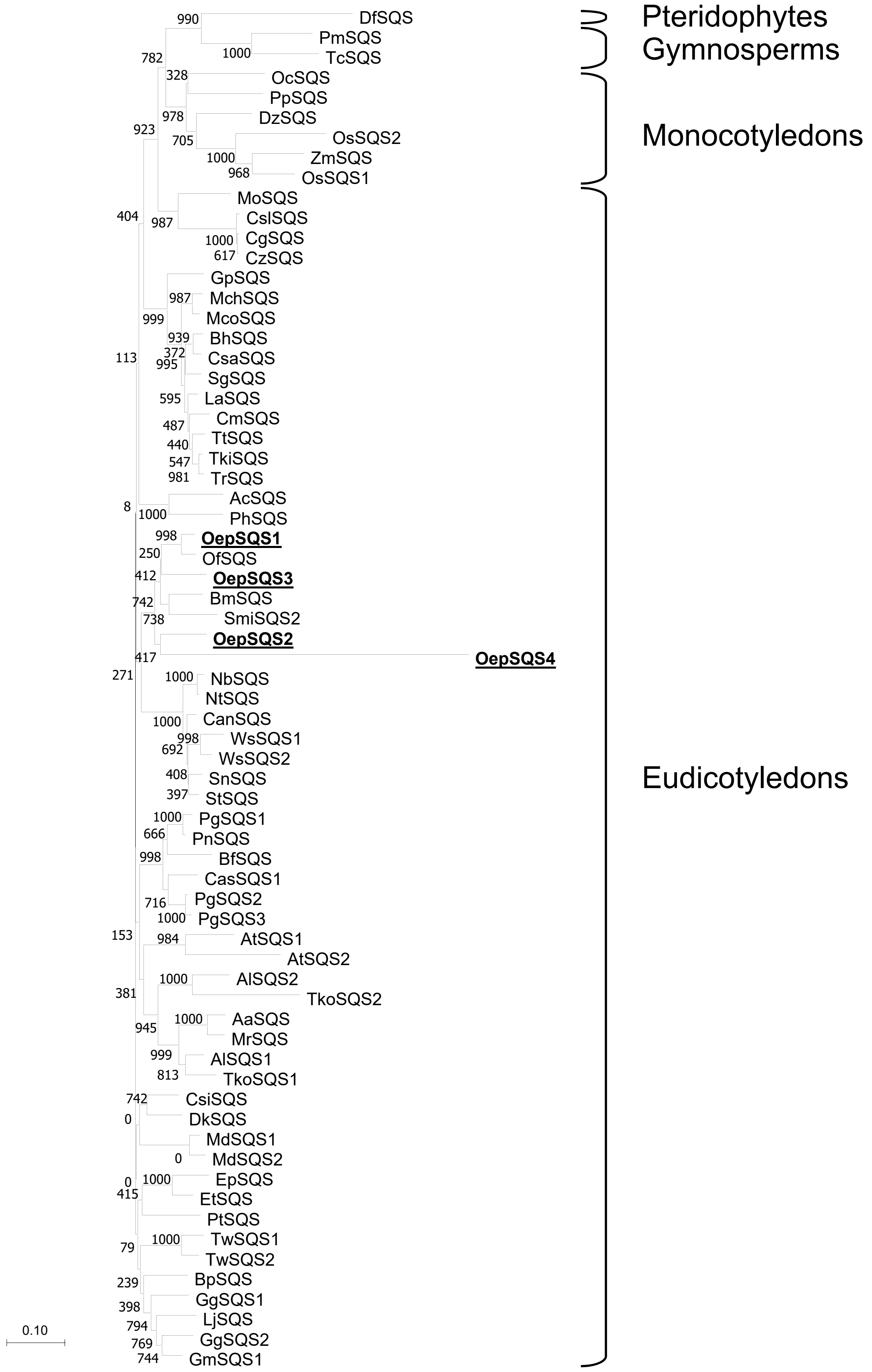


**Figure S3.** Phylogenetic tree analysis of plant squalene synthases. Alignments were calculated with ClustalX and the analysis was performed using the neighbour-joining method implemented in the Phylip package using Kimura's correction for multiple substitutions, and a 1000 bootstrap data set. TreeView was used to display the tree. Positions of the olive SQS are in bold and underlined. Accession numbers of the different SQS included in this analysis are indicated in Supplementary Table S3.

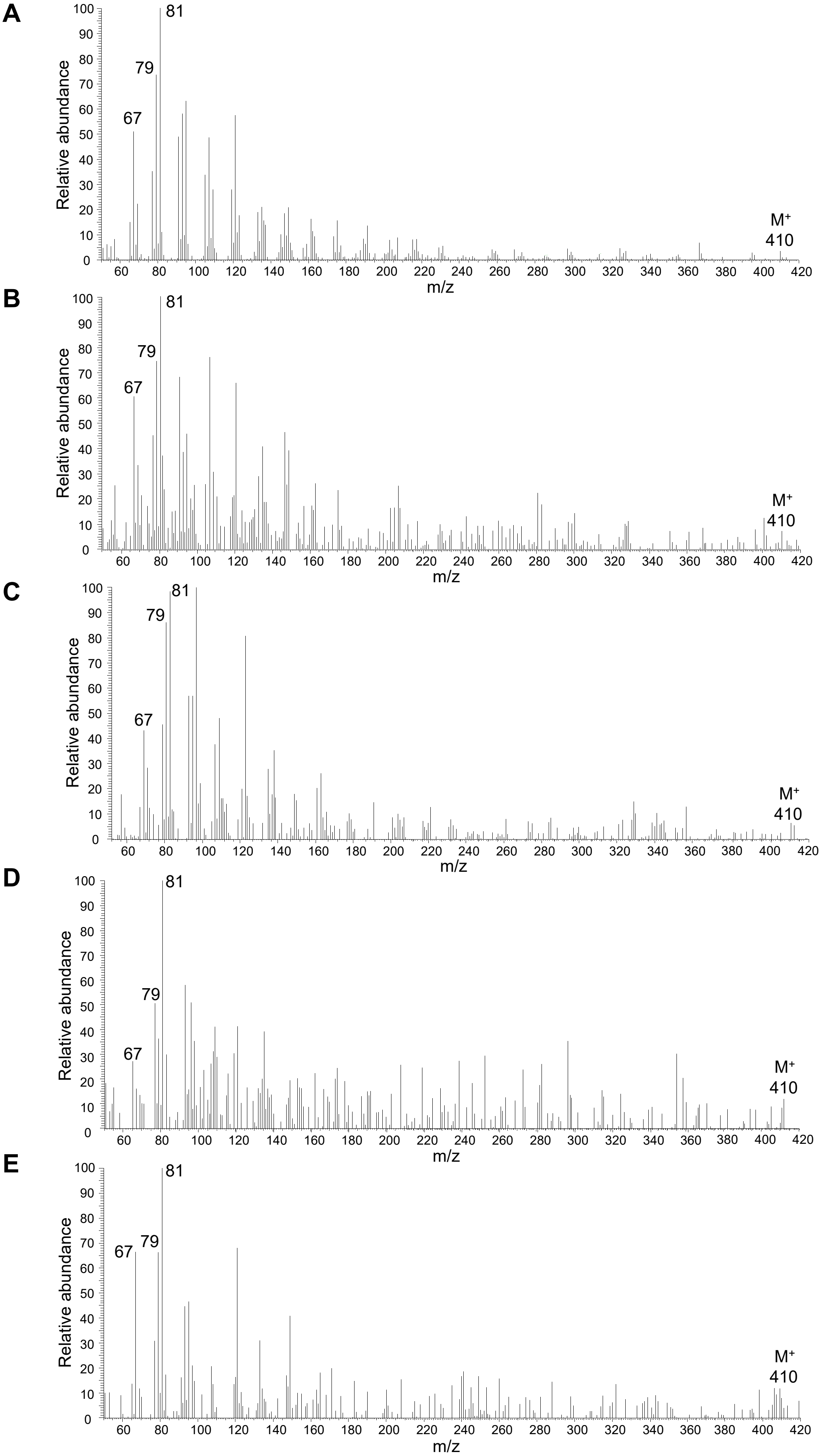


**Figure S4.** Mass spectrum of the peak for authentic squalene (A), and squalene synthesized by OepSQS1 (B), OepSQS2 (C), OepSQS3 (D) and OepSQS4 (E).

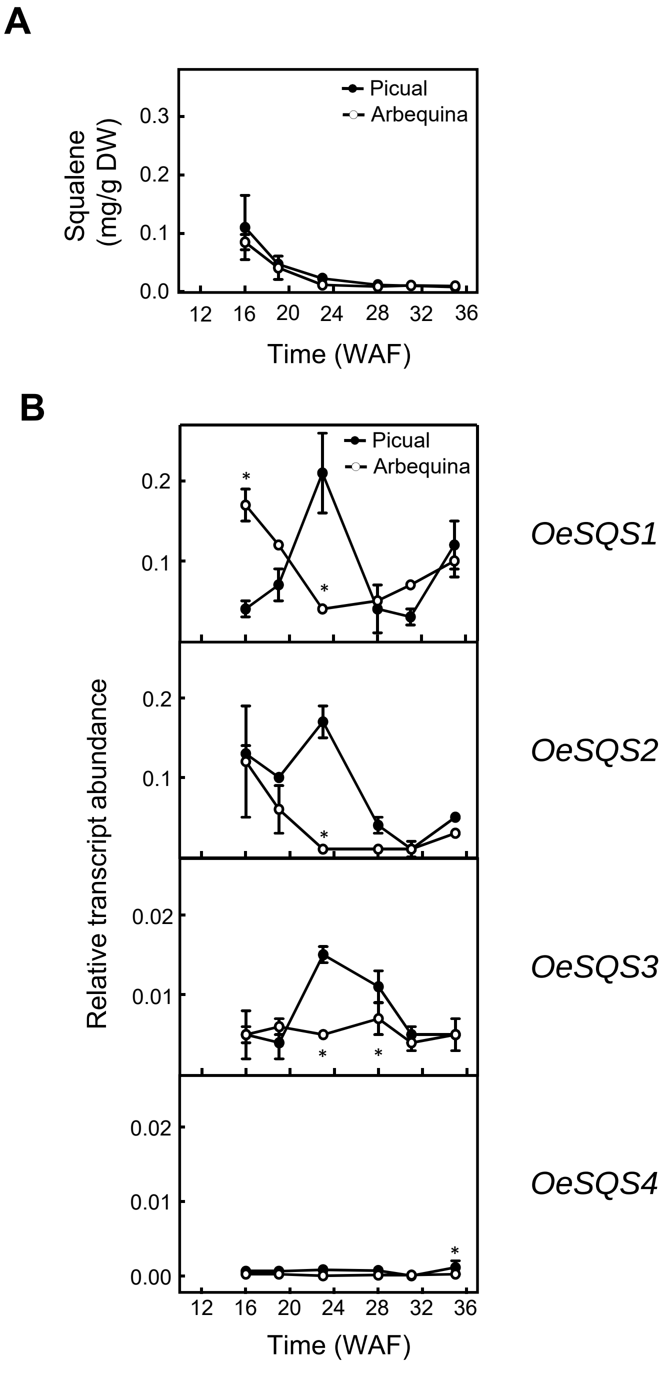


**Figure S5.** Squalene content (A) and relative transcript abundance of olive *SQS* genes (B) in the seed tissue of 'Picual' and 'Arbequina' cultivars during the olive fruit development and ripening. At the indicated times the amount of squalene was quantified by GC and the relative transcript abundance was determined by qRT-PC as described under Materials and methods. Data are presented as means ± SD of three biological replicates. *Indicates significantly different ($P$<0.05) to 'Picual' by two-way analysis of variance (ANOVA) with a Bonferroni posttest in 'Arbequina'.

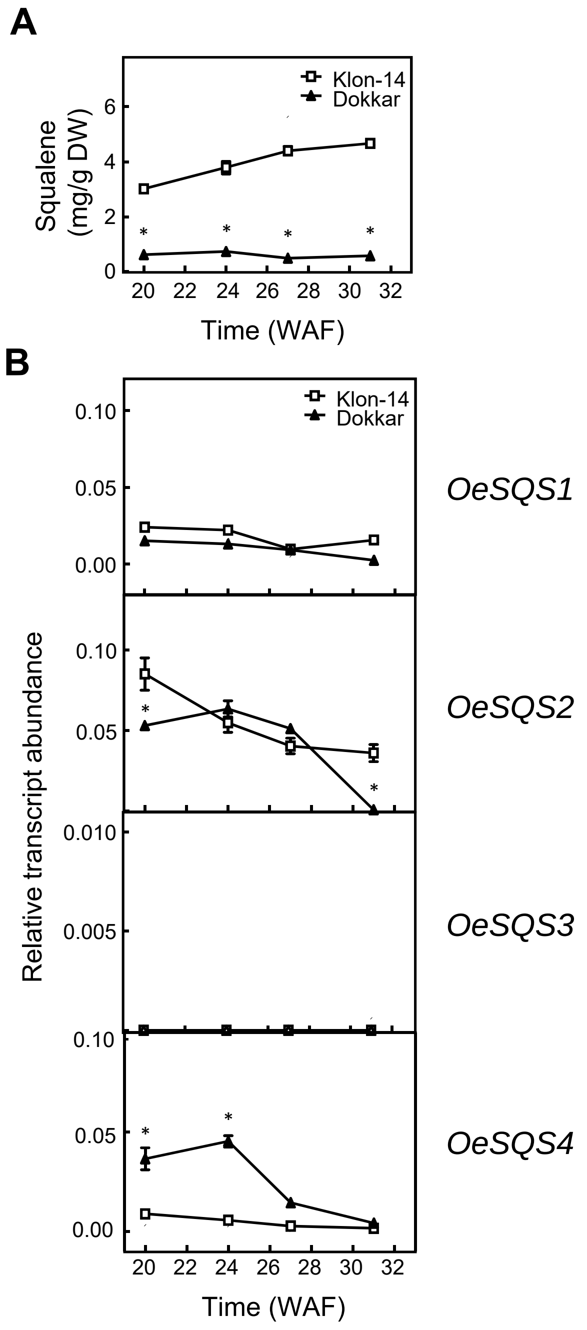


**Figure S6.** Squalene content (A) and relative transcript abundance of olive *SQS* genes (B) in mesocarp tissue during the olive fruit development and ripening of 'Dokkar' and 'Klon-14' cultivars. At the indicated times the amount of squalene was quantified by GC and the relative transcript abundance was determined by qRT-PC as described under Materials and methods. Data are presented as means ± SD of three biological replicates. *Indicates significantly different ($P$<0.05) to 'Klon-14' by two-way analysis of variance (ANOVA) with a Bonferroni posttest in 'Dokkar'.

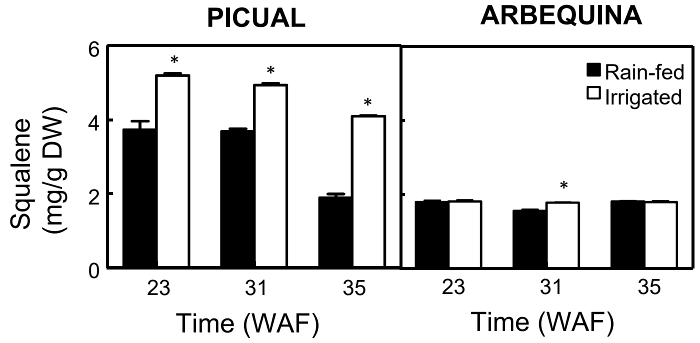


**Figure S7.** Effect of water regime on the squalene content of the mesocarp tissue from Picual and Arbequina cultivars during olive fruit development and ripening. At the indicated times the amount of squalene was quantified by GC as described under Materials and methods. Data are presented as means ± SD of three biological replicates. *Indicates significantly different (*P*<0.05) in irrigated to rain-fed conditions by two-way analysis of variance (ANOVA) with a Bonferroni posttest.

**Table S1.** Sequences of the primers pairs used for gene expression analysis by qRT-PCR.

| Gene | Sequence | Amplicon size (bp) |
|---|---|---|
| *OeSQS1* | Forward: 5'- GGAGCGATGAGCCTTTCTATC -3'<br>Reverse: 5'- CAGTGTTTCCAGTTGCTTTCC -3' | 119 |
| *OeSQS2* | Forward: 5'- GATTGCCGTCATCTTCATCAT -3'<br>Reverse: 5'- GCCCGCATTTTAACATTCTC -3' | 231 |
| *OeSQS3* | Forward: 5'- AAGCCAAGTTTTTCGAGTGTG -3'<br>Reverse: 5'- AATTCTTGAGGAAGGGCACTC -3' | 125 |
| *OeSQS4* | Forward: 5'- ATGACACCGAAACCACGAAG -3'<br>Reverse: 5'- CTTGCACAGCAGAAATGTCC -3' | 178 |
| *OeUBQ2* | Forward: 5'-AATGAAGTCTGTCTCTCCTTTGG-3'<br>Reverse: 5'-AAGGGAAATCCCATCAACG-3' | 132 |

**Table S2.** Sequences of the primers pairs used for amplification of olive *SQS* coding sequences for their functional expression in *E. coli*.

| Gene | Sequence | Restriction enzyme |
|---|---|---|
| *OepSQS1* | Forward: 5’- GAT<u>TTCGAA</u>**T**GGGAGTTTGAGGGCGATTTTG -3’ | *Bst*BI |
| | Reverse: 5’- ATC<u>TGTACA</u>TCATCTAGGCTCGCTCCTAATG -3’ | *Bsr*GI |
| *OepSQS2* | Forward: 5’- GAT<u>TTCGAA</u>**T**GGGAGTTTGAAGGCGATTT -3’ | *Bst*BI |
| | Reverse: 5’- ATC<u>TGTACA</u>TCATGTAGGCTCTTTCCTGATA -3’ | *Bsr*GI |
| *OepSQS3* | Forward: 5’- GAT<u>TTCGAA</u>**T**GGGAGTTTGAAGGCGATTTTG -3’ | *Bst*BI |
| | Reverse: 5’- TC<u>GGCGCGCC</u>TTATCTAGTCTTGGTCCTAATT -3’ | *Asc*I |
| *OepSQS4* | Forward: 5’- GAT<u>TTCGAA</u>**T**TCTAGAAATCCTCAACATG -3’ | *Bst*BI |
| | Reverse: 5’- ATC<u>TGTACA</u>TCACTCCTGATTATGTACT -3’ | *Bsr*GI |

Restriction sites are underlined. The nucleotides incorporated to maintain the reading frame of the genes are marked in bold.

**Table S3.** Accession numbers of the different plant SQS included in the phylogenetic tree analysis (Fig. S3).

| Plant | Gene | Accession Number |
|---|---|---|
| *Amaranthus cruentus* | *AcSQS* | AB691229 |
| *Arabidopsis thaliana* | *AtSQS1* | D29017 |
| *Arabidopsis thaliana* | *AtSQS2* | AT4G34650.1 |
| *Artemisia annua* | *AaSQS* | KP742985 |
| *Atractylodes lancea* | *AlSQS1* | MT740258 |
| *Atractylodes lancea* | *AlSQS2* | MT740259 |
| *Bacopa monniera* | *BmSQS* | GU734711 |
| *Benincasa hispida* | *BhSQS* | KX548335 |
| *Betula platyphylla* | *BpSQS* | KP723830 |
| *Bupleurum falcatum* | *BfSQS* | AY964186 |
| *Camellia sinensis* | *CsiSQS* | MF774226 |
| *Capsicum annuum* | *CanSQS* | AF124842 |
| *Centella asiatica* | *CasSQS* | AY787628 |
| *Chimonanthus grammalus* | *CgSQS* | MH277637 |
| *Chimonanthus salicifolius* | *CslSQS* | MH277638 |
| *Chimonanthus zhejiangensis* | *CzSQS* | MH277639 |
| *Cucumis sativus* | *CsaSQS* | KX548333 |
| *Cucurbita moscata* | *CmSQS* | KX548332 |
| *Dioscorea zingiberensis* | *DzSQS* | KC960673 |
| *Diospyros kaki* | *DkSQS* | FJ687954 |
| *Dryopteris fragrans* | *DfSQS* | KJ728647 |
| *Euphorbia pekinensis* | *EpSQS* | JX509735 |
| *Euphorbia tirucalli* | *EtSQS* | AB433916 |
| *Glycine max* | *GmSQS1* | AB007503 |
| *Glycyrrhiza glabra* | *GgSQS1* | D86409 |
| *Glycyrrhiza glabra* | *GgSQS2* | D86410 |
| *Gynostemma pentaphyllum* | *GpSQS* | FJ906799 |
| *Lotus japonicus* | *LjSQS* | AB102688 |
| *Luffa acutangula* | *LaSQS* | KX548336 |
| *Magnolia officinalis* | *MoSQS* | KT223496 |
| *Malus domestica* | *MdSQS1* | KC895979 |
| *Malus domestica* | *MdSQS2* | KC895980 |
| *Matricaria recutita* | *MrSQS* | KM279357 |
| *Momordica charantia* | *MchSQS* | KX548331 |
| *Momordica cochinchinensis* | *McoSQS* | KX548330 |
| *Nicotiana benthamiana* | *NbSQS* | U46000 |
| *Nicotiana tabacum* | *NtSQS* | U60057 |
| *Olea europaea* | *OepSQS1* | OQ676921 |
| *Olea europaea* | *OepSQS2* | OQ676922 |
| *Olea europaea* | *OepSQS3* | OQ676923 |
| *Olea europaea* | *OepSQS4* | OQ676924 |
| *Ornithogalum caudatum* | *OcSQS* | KX599231 |
| *Oryza sativa* | *OsSQS1* | AB007501 |
| *Oryza sativa* | *OsSQS2* | Os07g0200700 |
| *Osmanthus fragrans* | *OfSQS* | KY992860 |
| *Panax ginseng* | *PgSQS1* | AB010148 |
| *Panax ginseng* | *PgSQS2* | GQ468527 |

| | | |
|---|---|---|
| *Panax ginseng* | *PgSQS3* | GU183406 |
| *Panax notoginseng* | *PnSQS* | DQ186630 |
| *Paris polyphylla* | *PpSQS* | MZ274227 |
| *Pinus massoniana* | *PmSQS* | KF857540 |
| *Populus trichocarpa* | *PtSQS* | XM_002313729.3 |
| *Pseudostellaria heterophylla* | *PhSQS* | KY436585 |
| *Salvia miltiorrhiza* | *SmiSQS2* | KM408605 |
| *Siraitia grosvenorii* | *SgSQS* | KX231777 |
| *Solanum nigrum* | *SnSQS* | JX984610 |
| *Solanum tuberosum* | *StSQS* | AB022599 |
| *Taraxacum koksaghyz* | *TkoSQS1* | MG646369 |
| *Taraxacum koksaghyz* | *TkoSQS2* | MG646370 |
| *Taxus cuspidata* | *TcSQS* | DQ836053 |
| *Trichosanthes kirilowii* | *TkiSQS* | KX548329 |
| *Trichosanthes rubriflos* | *TrSQS* | KX548326 |
| *Trichosanthes truncata* | *TtSQS* | KX548327 |
| *Tripterygium wilfordii* | *TwSQS1* | KR401220 |
| *Tripterygium wilfordii* | *TwSQS2* | MN401748 |
| *Withania somnifera* | *WsSQS1* | GU474427 |
| *Withania somnifera* | *WsSQS2* | GU732820 |
| *Zea mays* | *ZmSQS* | AB007502 |
